# Efficient upgrading of CO to C_3_ fuel using asymmetric C-C coupling active sites

**DOI:** 10.1038/s41467-019-13190-6

**Published:** 2019-11-29

**Authors:** Xue Wang, Ziyun Wang, Tao-Tao Zhuang, Cao-Thang Dinh, Jun Li, Dae-Hyun Nam, Fengwang Li, Chun-Wei Huang, Chih-Shan Tan, Zitao Chen, Miaofang Chi, Christine M. Gabardo, Ali Seifitokaldani, Petar Todorović, Andrew Proppe, Yuanjie Pang, Ahmad R. Kirmani, Yuhang Wang, Alexander H. Ip, Lee J. Richter, Benjamin Scheffel, Aoni Xu, Shen-Chuan Lo, Shana O. Kelley, David Sinton, Edward H. Sargent

**Affiliations:** 1 Department of Electrical and Computer Engineering, University of Toronto, 35 St George Street, Toronto, ON M5S 1A4 Canada; 2 Department of Mechanical and Industrial Engineering, University of Toronto, 5 King’s College Road, Toronto, ON M5S 3G8 Canada; 3 Material and Chemical Research Laboratories, Industrial Technology Research Institute, Hsinchu, 31040 Taiwan; 4 Center for Nanophase Materials Sciences, Oak Ridge National Laboratory, 1 Bethel Valley Road, Oak Ridge, TN 37830 USA; 5 Department of Chemistry, University of Toronto, 80 St George Street, Toronto, ON M5S 3H6 Canada; 6 Materials Science and Engineering Division, National Institute of Standards and Technology (NIST), 100 Bureau Dr, Gaithersburg, MD 20899 USA; 7 Department of Pharmaceutical Sciences, Leslie Dan Faculty of Pharmacy, University of Toronto, 144 College Street, Toronto, ON M5S 3M2 Canada

**Keywords:** Electrocatalysis, Carbon capture and storage, Renewable energy, Materials for energy and catalysis

## Abstract

The electroreduction of C_1_ feedgas to high-energy-density fuels provides an attractive avenue to the storage of renewable electricity. Much progress has been made to improve selectivity to C_1_ and C_2_ products, however, the selectivity to desirable high-energy-density C_3_ products remains relatively low. We reason that C_3_ electrosynthesis relies on a higher-order reaction pathway that requires the formation of multiple carbon-carbon (C-C) bonds, and thus pursue a strategy explicitly designed to couple C_2_ with C_1_ intermediates. We develop an approach wherein neighboring copper atoms having distinct electronic structures interact with two adsorbates to catalyze an asymmetric reaction. We achieve a record *n*-propanol Faradaic efficiency (FE) of (33 ± 1)% with a conversion rate of (4.5 ± 0.1) mA cm^−2^, and a record *n*-propanol cathodic energy conversion efficiency (EE_cathodic half-cell_) of 21%. The FE and EE_cathodic half-cell_ represent a 1.3× improvement relative to previously-published CO-to-*n*-propanol electroreduction reports.

## Abstract (web summary)

Catalysts for CO electroreduction have focused on Cu, and their main products have been C_2_ chemicals. Here authors use the concept of asymmetric active sites to develop a class of doped Cu catalysts for C-C coupling, delivering record selectivity to *n*-propanol.

## Introduction

The CO_2_ electroreduction reaction (CO_2_RR) to high-energy-density liquid products is an attractive avenue to achieving the storage of renewable energy. In recent years, much progress has been made in CO_2_RR, but the main products reported have been C_1_ (CO, CH_4_, methanol, and formate) and C_2_ (ethylene, acetate, and ethanol) products^1–13^. *n*-propanol, a high-value and high-energy-density C_3_ product that can be generated via either CO_2_ or CO electroreduction^14,15^, has been produced with low-to-moderate Faradaic efficiencies (FEs) in prior reports^10,12,16–26^.

In CO_2_RR, the formation of multi-carbon products starts with the formation of the CO intermediate, followed by the CO electroreduction process^27–29^. Recently, significant progress has been made in CO_2_RR to CO with a FE nearly 100%^30–32^. To achieve the ultimate goal of high selectivity to high-value-added C_3_ products from CO_2_RR, it is of interest to improve significantly the FE for CO electroreduction (CORR) to C_3_ products.

The formation of C_3_ products from CORR relies on the sequential formation of two carbon–carbon (C–C) bonds, the main reaction mechanism for C_3_ formation reported previously^25,33,34^. Cu provides excellent C–C coupling and produces multi-carbon chemicals in the electroreduction of CO; however, the selectivity towards C_3_ products on Cu has remained low^35,36^. The generation of C_3_ products from CO requires multiple product/intermediate formation steps, and it is prone to the competing production of a wide variety of chemical products^33,34,37,38^.

Increasing selectivity in the electroreduction of CO to C_3_ products is thus an important challenge to address in the field of electrocatalysis. Until now, catalysts for CORR have focused on Cu and oxide-derived Cu catalysts, and a number of factors have been found to increase performance: these include engineering the oxidation state of the atoms making up the metal catalyst, as well as grain-boundary effects and the selective formation of desired facets^16,21–26,39^. However, the main products of these Cu and oxide-derived Cu catalysts have been C_2_ chemicals (ethanol, acetate, and ethylene), and the selectivity to C_3_ products has saturated in recent manuscripts based on Cu catalysts.

Here we explore instead a doping strategy involving different metal-doped Cu (*M*-doped Cu) catalysts in an attempt to increase C_3_ production in CORR. The low C_3_ selectivity on Cu catalysts is associated with the low rate of C–C bond formation, including C_1_–C_1_ and C_1_–C_2_ coupling. Mechanisms underpinning C_1_–C_1_ coupling to C_2_ products have been explored extensively in prior studies^29,33,38,40–42^; while C_1_–C_2_ coupling to C_3_ products is less explored. Here we screen the propensity to catalyze C_1_–C_1_ and C_1_–C_2_ coupling using density functional theory (DFT) based on M-doped Cu catalysts. We find that, among different *M*-doped Cu candidates explored, Ag-doped Cu is expected to offer the highest activity for both C_1_–C_1_ and C_1_–C_2_ coupling, and we pinpoint a role for the asymmetric C–C coupling active site in this high activity. Specifically, this active site consists of two neighboring Cu atoms that exhibit different electronic structures: this asymmetry among the neighbors’ energetics is determined by the combination of strain and ligand effects arising upon Ag doping. We then fabricate Ag-doped Cu nanocatalysts via a galvanic replacement approach. We demonstrate that the synthesized Ag-doped Cu catalyst exhibits higher FEs for *n*-propanol compared to all previous CORR reports^10,12,16–26^. This leads to superior energy conversion efficiency in the cathodic half-cell (EE_cathodic half-cell_) for *n*-propanol.

## Results

### Computational modeling and catalyst design principles

**Fig. 1:**
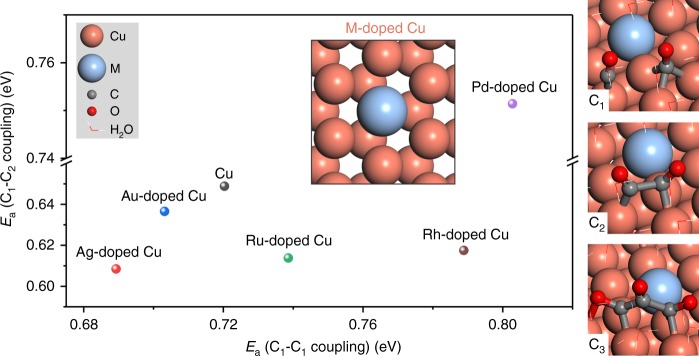
DFT calculations on C_1_–C_1_ and C_1_–C_2_ coupling. DFT calculated reaction barriers (*E*_a_) for C_1_–C_1_ and C_1_–C_2_ coupling on screened *M*-doped Cu systems (*M* = Ag, Au, Ru, Rh, and Pd). The geometries of *M*-doped Cu surface, C_1_, C_2_, and C_3_ on *M*-doped Cu are shown with the corresponding labels, respectively. Cu, M, C, and O are illustrated as orange, light blue, gray, and red balls, respectively, while water molecules are shown as lines

Catalysts that convert CO into C_3_ chemicals require high activity for both C_1_–C_1_ and C_1_–C_2_ coupling. With the goal of designing better catalysts for C_3_ production, we investigated the energetics of C_1_–C_1_ and C_1_–C_2_ coupling reactions to illustrate the C_2_ and C_3_ formation rates with the aid of DFT (more details of DFT methods and choice of sequential mechanism can be found in Supplementary Information). Several M-doped Cu systems (*M* = Ag, Au, Ru, Rh, and Pd) were considered because bimetallic catalysts have been shown to tune catalyst performance in other catalytic reactions^43,44^. CO dimerization is one reaction pathway for C_1_–C_1_ coupling^29,40^, so we used the barrier of CO dimerization to describe the readiness of C_1_–C_1_ coupling. Due to the abundance of CO species in CORR, we used the barrier of OCCO and CO coupling as the indicator for the C_1_–C_2_ coupling (Supplementary Figs. 1–14 and Supplementary Tables 1–3). As shown in Fig. 1a, among the *M*-doped Cu systems studied, calculation results show that Ag-doped Cu possesses the lowest activation energies for both C_1_–C_1_ and C_1_–C_2_ coupling, suggesting that Ag-doped Cu is a promising catalyst for the formation of C_3_ products from CO.

We carried out further theoretical investigations to uncover the physical origins of enhancement in C_1_–C_1_ and C_1_–C_2_ coupling on Ag-doped Cu. We used a model with one Ag atom doped in a Cu(111) slab with a 3 × 3 unit cell, which corresponds to about 3% doping concentration for four layers of Cu atoms. By a margin of 0.63 eV, Ag at the surface of Cu(111) is more favorable than in subsurface of Cu(111), inducing us to focus on surface-localized Ag in our ensuing studies.

As the radius of the Ag atom is larger than that of Cu atom, Ag doping produces surface strain. The bond length of Cu–Cu changed from 2.57 Å on the Cu(111) surface to 2.55 and 2.48 Å on the Ag-doped Cu surface, resulting in asymmetric compressive strain (Supplementary Fig. 15). The ligand effect^45^ caused by Ag doping in Cu also has the potential to affect C_1_–C_1_ and C_1_–C_2_ coupling. To evaluate the effect of the strain and ligand, we built a model with the same strain of Ag-doped Cu but without Ag substitution by fixing the bond length of Cu surface (denoted *Cu with strain*). We calculated the activation energies for C_1_–C_1_ and C_1_–C_2_ coupling steps on Cu, Cu with strain, and Ag-doped Cu models (Supplementary Table 4). Calculation results show that both compressive strain and ligand effects contribute to enhanced activity for C_1_–C_1_ and C_1_–C_2_ coupling (Supplementary Fig. 16a).

Ag doping in Cu leads to two classes of neighboring Cu atoms (denoted Cu-a and Cu-b atoms, Supplementary Fig. 16b). The two classes exhibit distinct electronic structures, as a result of strain and ligand effects. As to the strain effect, the bond length between Cu-a and Cu-b atoms is 2.55 Å, while the bond length between two Cu-b atoms is 2.48 Å (Supplementary Fig. 15), suggesting that Cu-a is more isolated than Cu-b. As to ligand effects, Cu-a coordinates with nine Cu atoms, while Cu-b coordinates with eight Cu atoms and one Ag atom (Supplementary Fig. 16). We term a pair of adjacent Cu atoms with different electronic structures an *asymmetric C–C coupling active site*. In this reaction, the asymmetric site interacts with two CO to yield asymmetric reactants—two adsorbed CO on Cu-a and Cu-b atoms with different electronic structures—enhancing C_1_–C_1_ coupling. In light of the similarity with C–C coupling, the same site can further promote C_1_–C_2_ coupling between asymmetric C_1_ and C_2_ intermediates (Supplementary Fig. 17). These findings are in ways analogous to the enhanced coupling effect of Cu^0^ and Cu^+^ proposed by Goddard and co-workers^41^. Taken together, these DFT calculation results suggest that Ag-doped Cu with asymmetric C–C coupling active sites is a good candidate for C_3_ production in CORR which appear to support both C_1_–C_1_ dimerization and C_1_–C_2_ coupling. It is worth mentioning that there exist other C_1_–C_2_ coupling possibilities: we also examined another C_1_–C_2_ coupling mechanism, OC-OCCOH (refs. ^33,39^), and found that Ag-doped Cu had a barrier of 0.76 eV, lower than 0.88 eV on Cu (Supplementary Figs. 18, 19, and Supplementary Table 5).

### Preparation and characterization of nanocatalysts

**Fig. 2:**
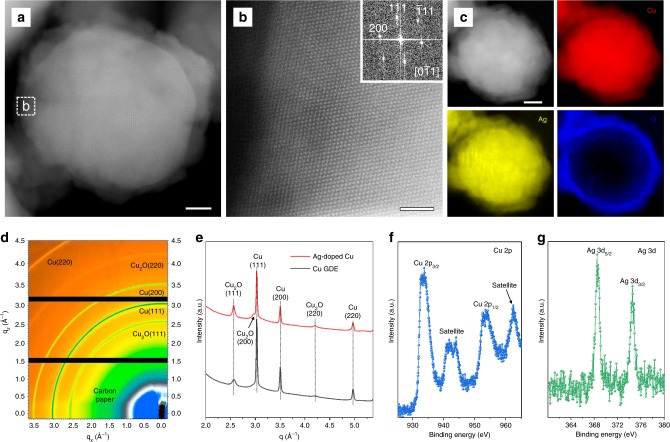
Structural and compositional analyses of Ag-doped Cu catalyst. **a** High-angle annular dark-field scanning transmission electron microscopy (HAADF-STEM) image taken from a single particle. Scale bar, 20 nm. **b** Atomic-resolution HAADF-STEM image taken from the edge of a nanoparticle marked by a box in (**a**). Inset, the corresponding Fourier transfer image. Scale bar, 2 nm. **c** HAADF-STEM image of an Ag-doped Cu nanoparticle and the corresponding EELS elemental mappings of Cu, Ag, and O. Scale bar, 20 nm. **d**,**e** WAXS map (**d**) and the corresponding sector-average of WAXS map (**e**) for Ag-doped Cu GDE. **f**, **g** High-resolution Cu 2p (**f**) and Ag 3d (**g**) spectra of Ag-doped Cu GDE

We sought to prepare experimentally Ag-doped Cu catalysts. We employed a galvanic replacement reaction driven by the difference in the reduction potential of Ag vs. Cu (ref. ^46^). Firstly, we deposited a thin layer of commercial Cu nanoparticles with average size of 100 nm on a carbon-based gas diffusion layer (GDL) via spray-coating (Supplementary Fig. 20a). The Cu gas diffusion electrode (GDE) was then immersed in N_2_-saturated 5 μmol L^−1^ AgNO_3_ aqueous solution at 65 °C for 1 h to obtain the Ag-doped Cu GDE. The Ag-doped Cu catalyst retains the particle size and the morphology of the pristine Cu nanoparticles (Fig. 2a, b, and Supplementary Figs. 20 and 21). Electron energy loss spectroscopy (EELS) elemental mapping showed that Ag and Cu elements were uniformly distributed in the particle (Fig. 2c and Supplementary Fig. 22). In transmission wide angle X-ray scattering (WAXS) data and powder X-ray diffraction (XRD) patterns, we observed peaks for Cu_2_O in both Cu and Ag-doped Cu GDE (Fig. 2d, e, and Supplementary Fig. 23), which were attributed to oxidation of Cu in air during the preparation of GDE. Due to the low concentration of Ag (4.0% atomic fraction determined by XPS), a peak shift relative to Cu is not observed in WAXS data of the Ag-doped Cu GDE (Fig. 2e and Supplementary Table 6). X-ray photoelectron spectroscopy (XPS) of the Ag-doped Cu GDE also showed the existence of CuO on the surface and confirmed the presence of Ag^0^ (Fig. 2f, g).

### CORR performance and Operando X-ray absorption spectroscopy

**Fig. 3:**
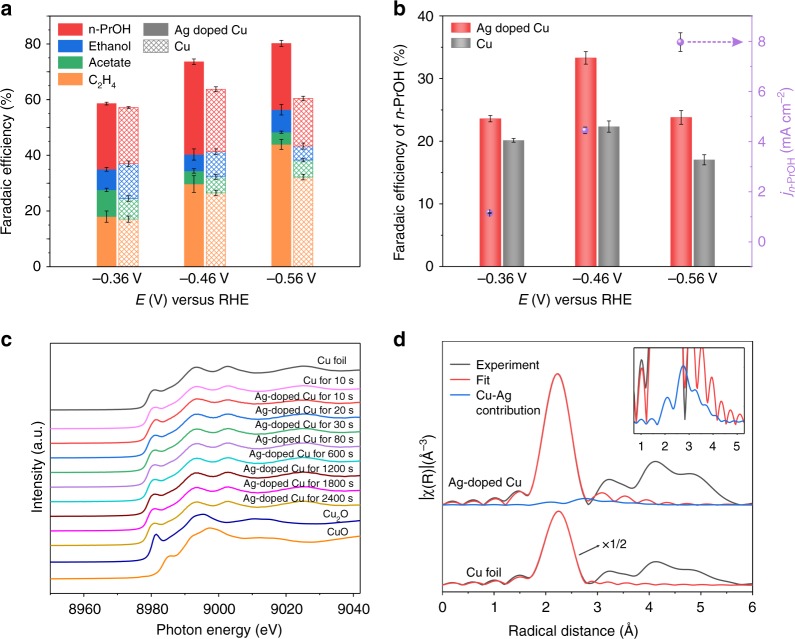
CO electroreduction performance and operando structural characterizations of Ag-doped Cu catalyst in flow cell. **a** FEs of *n*-propanol (*n*-PrOH), ethanol, acetate, and ethylene on Ag-doped Cu and pristine Cu catalysts under different potentials. **b** Comparison of *n*-propanol FEs on Ag-doped Cu and Cu GDE under different potentials, as well as partial current density of *n*-propanol formation on Ag-doped Cu GDE. Error bars represent the standard deviation based on three separate measurements. **c** Operando Cu K-edge XANES spectra of Cu GDE following 10 s and Ag-doped Cu GDE following 10, 20, 30, 80, 600, 1200, 1800, and 2400 s at −0.46 V_RHE_ during CORR. Bulk Cu foil, CuO, and Cu_2_O are listed as references. **d** Operando Cu K-edge EXAFS spectrum of Ag-doped Cu GDE following 10 s at −0.46 V_RHE_ during CORR. Bulk Cu foil was listed as a reference. Inset, an enlarged view of the section from 0.7 to 5.3 Å for Ag-doped Cu GDE

We then investigated the performance of the Ag-doped Cu GDE in CORR flow cell reactors (Supplementary Fig. 24). Flow cells overcome the mass transfer limitation of CO and produce a triple-phase interface that allows the gas reactant to contact the catalyst-electrolyte interface during the reaction. Figure 3a shows FEs for C_2+_ products in the applied potential range of −0.36 to −0.56 V with reference to the reversible hydrogen electrode (RHE) in 1 M KOH electrolyte. The liquid products (*n*-propanol, ethanol, and acetate) and gas products (ethylene and H_2_) were quantified using nuclear magnetic resonance (NMR) and gas chromatography, respectively (Supplementary Fig. 25 and Supplementary Table 7). In the applied potential range of −0.36 to −0.56 V_RHE_, the total C_2+_ FEs on Ag-doped Cu GDE are higher than that on Cu GDE: indeed the total FE of C_2+_ products on Ag-doped Cu GDE reaches about 80% at -0.56 V_RHE_. In particular, at a low potential of −0.46 V_RHE_, the Ag-doped Cu GDE records a high *n*-propanol FE of (33 ± 1)% with the partial *n*-propanol current density of (4.5 ± 0.1) mA cm^-2^, whereas *n*-propanol FE on pristine Cu is (22 ± 1)% (Fig. 3b). This impressive *n*-propanol FE represents the highest value reported for *n*-propanol production via CO_2_RR and CORR (Supplementary Table 8). The higher FE for C_2+_ and C_3_ products on Ag-doped Cu relative to Cu is consistent with predictions from DFT. The intrinsic activities for *n*-propanol production on Ag-doped Cu and Cu are reported via the partial current density for *n*-propanol production normalized to the electrochemical surface area (ECSA) (Supplementary Figs. 26, 27, and Supplementary Table 9). The ECSA-normalized partial *n*-propanol current density on Ag-doped Cu is 0.124 mA cm^-2^, which is 3 times that on Cu. The *n*-propanol EE_cathodic half-cell_ reaches 20% at a low potential of −0.46 V_RHE_ when the overpotential of oxygen evolution in anode side is assumed to be 0. After correcting for ohmic loss (Supplementary Table 10), the *n*-propanol EE_cathodic half-cell_ reaches 21% under a low overpotential of 0.616 V. This EE_cathodic half-cell_ is higher than the best prior reports by a margin of 1.3× (Supplementary Sections 3, 4, and Supplementary Table 8).

It should be noted that the highest *n*-propanol FE on both Ag-doped Cu and Cu GDEs were achieved at relatively low potential (-0.46 V_RHE_), and *n*-propanol FE decreased when further increasing the potential to −0.56 V_RHE_ (Fig. 3a and Supplementary Table 7). The total C_2_ product and ethylene FEs on both Ag-doped Cu and Cu GDEs exhibited an increasing trend with increased applied potentials. This result can be explained by noting that the C–C coupling step for *n*-propanol formation becomes slow at high potential, and thus C_2_ intermediate protonation reaction is more favored compared with C–C coupling step for *n*-propanol formation^26,34^.

To study the chemical state of the catalyst during CORR, we performed operando X-ray absorption spectroscopy (XAS) of Ag-doped Cu GDE under operando CORR conditions at a constant applied potential of −0.46 V_RHE_. Operando Cu K-edge X-ray absorption near edge structure (XANES) spectra of Ag-doped Cu GDE show that Cu atoms were reduced to Cu^0^ in the first 10 s during CORR (Fig. 3c and Supplementary Fig. 28). Thereafter, the valence state of Cu is maintained at zero throughout CORR. Consistent results are shown in the operando extended X-ray adsorption fine structure (EXAFS). An EXAFS fitting analysis at the Cu K-edge showed the presence of Ag and its strong interaction with Cu by having Cu–Ag distance value between pure Cu and pure Ag (Fig. 3d and Supplementary Table 11)^13^. Collectively, operando XAS results demonstrated that Ag atoms are doped in the lattice of Cu nanoparticles, and that each element remains in its metallic state during CORR. As a control experiment, we carried out operando Cu K-edge XANES on Cu GDE, and it also showed that Cu oxides were reduced to Cu^0^ in the first 10 s during CORR. These results further demonstrated that, rather than benefiting from oxidation states^8,13,16,21,47^, the high FE of *n*-propanol on Ag-doped Cu GDE is associated with metallic states of Cu and might be ascribed to the different structures of Ag-doped Cu relative to Cu.

**Fig. 4:**
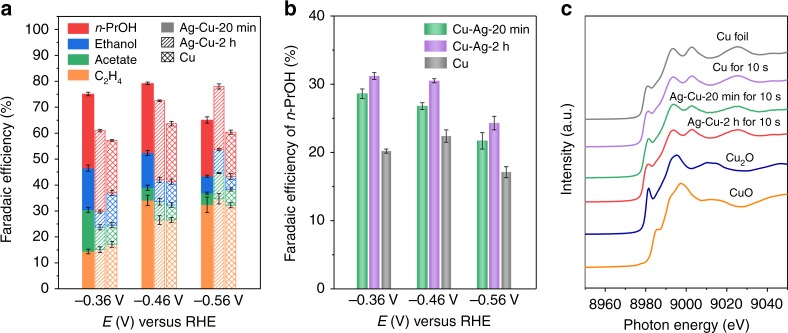
CO electroreduction performance on different types of Ag-doped Cu catalysts and operando structural characterizations in flow cell. **a** FEs of *n*-propanol, ethanol, acetate, and ethylene on Cu–Ag-20 min, Cu–Ag-2 h, and Cu GDE under different potentials. **b** Comparison of *n*-propanol FEs on Cu–Ag-20 min, Cu–Ag-2 h, and commercial Cu GDE. Error bars represent the standard deviation based on three separate measurements. **c** Operando Cu K-edge XANES spectra of Cu–Ag-20 min, Cu–Ag-2 h, and Cu GDE following 10 s at −0.46 V_RHE_. Bulk Cu foil, Cu_2_O, and CuO are listed as references

To explore further the role of Ag doping in Cu in facilitating C_1_–C_1_ and C_1_–C_2_ coupling, we investigated the CORR performance of Ag-doped Cu catalysts with different Ag concentrations. To vary the Ag concentration, we changed the immersion time of the Cu GDE in AgNO_3_ solution to 20 min and 2 h, respectively, denoted Cu–Ag-20 min and Cu–Ag-2 h. As in Ag-doped Cu, both Cu–Ag-20 min and Cu–Ag-2 h catalysts also retained the particle size and the morphology of the pristine Cu nanoparticles (Supplementary Figs. 29–31). Thus, the size and morphology effect can be excluded when comparing the CORR performance between Cu and different types of Ag-doped Cu catalysts. The atomic percentages of Ag doping in Cu were tuned to 2.4% and 7.8% (XPS), respectively, for Cu–Ag-20 min and Cu–Ag-2 h catalysts (Supplementary Figs. 32, 33, and Supplementary Table 6). Relative to Cu GDE, both Cu–Ag-20 min and Cu–Ag-2 h GDE exhibited enhanced FEs of *n*-propanol, as well as higher FEs of total C_2+_ products (Fig. 4a, b), in the potential range from −0.36 to −0.56 V_RHE_ toward CORR. These results are in agreement with DFT predictions of enhancement in C_1_–C_1_ and C_1_–C_2_ coupling by Ag doping in Cu. Additionally, among Ag-doped Cu catalysts with different Ag concentrations, Ag-doped Cu with an atomic percentage of 4.0% Ag showed the highest *n*-propanol FE at -0.46 V_RHE_ (Supplementary Fig. 34). The analysis of operando Cu K-edge XANES of Cu–Ag-20 min and Cu–Ag-2 h GDE also demonstrates that Cu oxides are reduced to Cu^0^ in the first 10 s during CORR (Fig. 4c) and it is Ag-doped Cu in metallic state that is associated with the enhanced selectivity to *n*-propanol during CORR.

The carbon-based GDLs suffer from liquid penetration and gas diffusion blockage, termed flooding, over time. To overcome the flooding issue on carbon-based GDE after long operation time, we fabricated the Ag-doped Cu polytetrafluoroethylene (PTFE) electrode based on a configuration (graphite/carbon nanoparticle/Ag-doped Cu/PTFE electrode) developed by our group^7^. The Ag-doped Cu layer was prepared by immersing a Cu layer in 5 μmol L^−^^1^ AgNO_3_ aqueous solution at 65 °C for 1 h. When a potential of −0.46 V_RHE_ was applied, the FE of *n*-propanol on the Ag-doped Cu PTFE electrode achieved 33% and operated stably over 200 min of CORR (Supplementary Figs. 35 and 36).

We also compared the CO_2_RR performance of Ag-doped Cu and pristine Cu to explore whether the asymmetric active sites enhance in this distinct context the C_1_–C_1_ and C_1_–C_2_ coupling (Supplementary Fig. 37 and Supplementary Table 12). In the potential range from −0.82 to −1.79 V_RHE_ (after *i*R compensation), both C_2+_ and C_3_ FEs on Ag-doped Cu catalysts are notably higher than those on pristine Cu: at the potential of −2.96 V_RHE_ (−1.31  V_RHE_ after *i*R compensation), the partial C_2+_ and *n*-propanol current densities of Ag-doped Cu are 308 ± 6 mA cm^−^^2^ and 36 ± 2 mA cm^−^^2^, and C_2+_ and *n*-propanol FEs on Ag-doped Cu are 62 and 7%, respectively, providing a doubling compared to pristine Cu.

## Discussion

This work demonstrates Ag doping in Cu to facilitate C_1_–C_1_ and C_1_–C_2_ coupling and thus improve the selectivity to C_3_ products during CORR. DFT results show that the strain and ligand effects due to Ag doping jointly provide an asymmetric C–C coupling active site containing two neighboring Cu atoms with different electronic structures, and that these are capable of enhancing C_1_–C_1_ and C_1_–C_2_ coupling. Experimentally, we achieved a total C_2+_ FE of about 80% and a record *n*-propanol FE of (33 ± 1)% with a partial *n*-propanol current density (4.5 ± 0.1) mA cm^-2^ on Ag-doped Cu catalyst in CORR. The EE_cathodic half-cell_ for *n*-propanol also reaches 21% at a low potential of 0.416  V_RHE_, with a low overpotential of 0.616 V. These findings provide a framework for rational catalyst design for tuning the CORR selectivity towards high-energy-density C_3_ liquid products, a crucial step in overcoming the bottleneck of the electroproduction of C_3_ products.

## Methods

### DFT calculations

In this work, all the DFT calculations were carried out with a periodic slab model using the Vienna ab initio simulation program (VASP) (https://www.vasp.at/). Detailed theoretical methods are found in the Supplementary Information.

### Preparation of electrodes

A commercial Cu of 8.5 mg was dispersed in a mixture of 0.85 mL of methanol and 8.5 μL of 5% Nafion under ultrasonication for 30 min. The suspension was deposited on a carbon-based GDL using spray-coating with a catalyst loading of ≈1 mg cm^−^^2^ to prepare the Cu GDE. We then immersed the prepared Cu GDE in 5 μmol L^−^^1^ AgNO_3_ aqueous solution at 65 °C for a certain time period to prepare Ag-doped Cu GDE as cathodes. The main goal of the work was to focus on improving the efficiency of the cathodic side of CORR to propanol. Thus, we used Ni foam (1.6 mm thickness, MTI Corporation) as the oxygen evolution reaction (OER) catalyst in the anode side because it is commercially available and have been showed as a good OER catalyst^48^. Details of chemicals and materials information are found in the Supplementary Information.

### Characterization

Scanning electron microscopy (SEM) images were taken using a Quanta FEG 250 microscope (Certain commercial equipment, instruments, or materials are identified in this paper and Supplementary Information in order to specify the experimental procedure adequately. Such identification is not intended to imply recommendation or endorsement by the National Institute of Standards and Technology, nor is it intended to imply that the materials or equipment identified are necessarily the best available for the purpose). HAADF-STEM images were taken using an aberration-corrected FEI Titan 80–300 kV TEM/STEM microscope at 300 kV, with a probe convergence angle of 30 mrad and a large inner collection angle of 65 mrad to provide a nominal image solution of 0.7 Å. EELS elemental mapping was collected on aberration-corrected JEOL JEM-ARM200F electron microscope at 200 kV equipped with Gatan GIF quantum energy filters. Structural characterization of cathodes was obtained using XRD (MiniFlex600) with Cu-Kα radiation. The surface compositions of cathodes were determined by XPS (model 5600, Perkin-Elmer) using a monochromatic aluminum X-ray source. Operando XAS measurements were conducted at 9BM beamline at Advanced Photon Source (APS, Argonne national laboratory, IL). Athena and Artemis software included in a standard IFEFFIT package were used to process XAS data^49^. WAXS measurements were carried out in transmission geometry at the CMS beamline of the National Synchrotron Light Source II (NSLS-II), a U.S. Department of Energy (DOE) office of the Science User Facility operated for the DOE Office of Science by Brookhaven National Laboratory. Samples were measured with an imaging detector at a distance of 0.177 m using X-ray wavelength of 0.729 Å. Nika software package was used to sector average the 2D WAXS images^50^. Data plotting was done in Igor Pro (Wavemetrics, Inc., Lake Oswego, OR, USA).

### Electrochemical measurements

All the electrochemical measurements were conducted in flow cell reactor. Electrocatalytic measurements were operated using the three-electrode system at an electrochemical station (AUT50783). In the flow cell reactor, the prepared GDEs, anion exchange membrane, and nickel foam were positioned and clamped together between silicone gaskets and PTFE flow fields. Then 10 mL of electrolyte (1 M KOH aqueous solution) was introduced into the anode chamber between anode and membrane, as well as the cathode chamber between membrane and cathode, respectively. The electrolytes in cathode and anode were circulated by two pumps at the rate of 10 mL min^−^^1^. CO gas (Linde, 99.99%) or CO_2_ gas (Linde, 99.99%) was continuously supplied to gas chamber located at the back side of cathode GDE at the rate of 50 mL min^−^^1^. Gas could diffuse into the interface between cathode and electrolyte, thus generating a triple-phase interface between gas, electrode, and electrolyte. The catalytic performance of cathodes was evaluated by performing potentiostatic electrolysis.

All potentials were measured against an Ag/AgCl reference electrode (3 M KCl, BASi). Gas and liquid products were respectively analyzed using gas chromatograph (PerkinElmer Clarus 600) equipped with thermal conductivity and flame ionization detectors, and NMR spectrometer (Agilent DD2 600 MHz) by taking dimethylsulfoxide (DMSO) as an internal standard. All the potentials were converted to values with reference to RHE using $E_{{\rm{RHE}}} = E_{{\rm{Ag}}/{\rm{AgCl}}} + 0.210\;{\rm{V}} + 0.0591 \times {\rm{pH}}.$

ECSA was determined based on the equation ECSA = *R*_f_*S*, where *R*_f_ was roughness factor and *S* was geometric area of electrode (1 cm^−2^). *R*_f_ = *C*_dl_/29 μF cm^−2^, where *C*_dl_ is the double-layer capacitance of catalyst and the double-layer capacitance of a smooth Cu surface is assumed to be 29 μF cm^−2^ (ref. ^21^). Double-layer capacitances of catalysts were determined by measuring cyclic voltammetry (CV) with different scan rates (40, 60, 80, 100, 120, and 140 mV s^−1^, respectively) in the potential ranges between 0.20 and 0.24 V_RHE_ where no Faradaic process occurred. The CV measurement was operated in the same flow cell reactor and 1 M KOH aqueous solution saturated with nitrogen (Linde, 99.998%) was used as the electrolyte. The flow cell reactor was filled with electrolyte prior to the CV measurement and the electrolyte was not circulated during the CV measurement. N_2_, instead of CO_2_, was continuously supplied to gas chamber of the cell. By plotting the average current *j* (*j* = (*j*_a_−*j*_c_)/2, where *j*_a_ and *j*_c_ are anodic and cathodic current densities, respectively) at 0.22 V_RHE_ against the scan rate, *C*_dl_ value was given by the slope.

The electrochemical impedance spectroscopy (EIS) technique was used to measure the ohmic loss between the working and reference electrodes and 70% *i*R compensation was applied to correct the potentials manually.

## Supplementary information

Supplementary Information

## Data Availability

The data supporting this study are available in the paper and the Supplementary Information. All other relevant source data are available from the corresponding authors upon reasonable request.

## Supplementary information

**Supplementary information** is avaliable for this paper at 10.1038/s41467-019-13190-6.
