# Supplementary material for: Efficient upgrading of CO to C3 fuel using asymmetric C-C coupling active sites
Source: Nat Commun. 2019 Nov 29;10:5186. doi: 10.1038/s41467-019-13190-6 (PMC6882816; doi:10.1038/s41467-019-13190-6)
Supplement: Supplementary file 1 — Supplementary Information [file 41467_2019_13190_MOESM1_ESM.pdf]

Efficient upgrading of CO to C_3 fuel using asymmetric C-C coupling active sites

Wang et al.

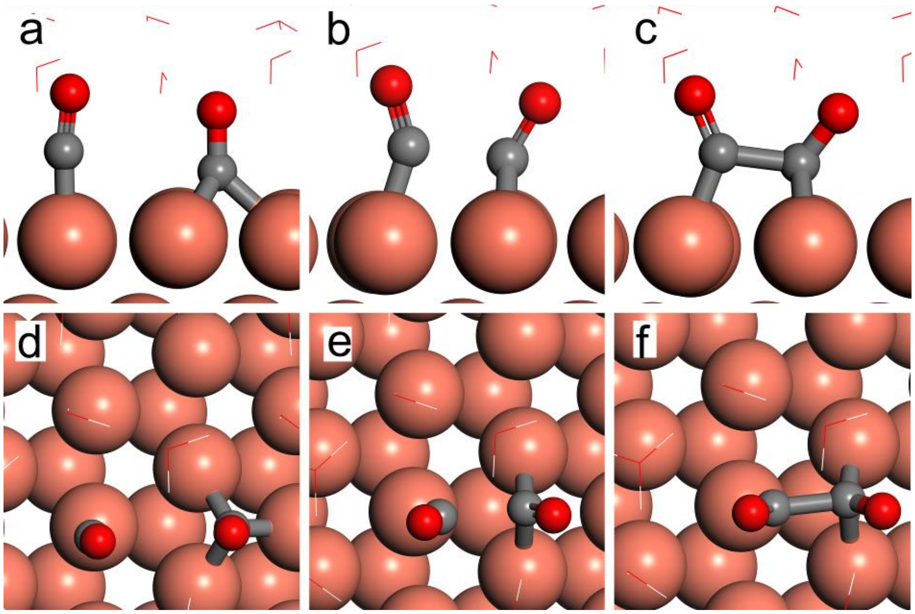

Supplementary Figure 1 | Geometries of CO dimerization on Cu(111) surface. a-c, Side views of initial state (**a**), transition state (**b**), and final state (**c**). **d-f,** Top views of initial state (**d**), transition state (**e**), and final state (**f**). The distance of CO molecules at the transition state of CO dimerization on Cu(111) is 1.897 Å. Red, grey, and light red balls stand for oxygen, carbon and copper atoms, respectively. Water molecules are shown as lines. These notations are used throughout in the Supplementary Information.

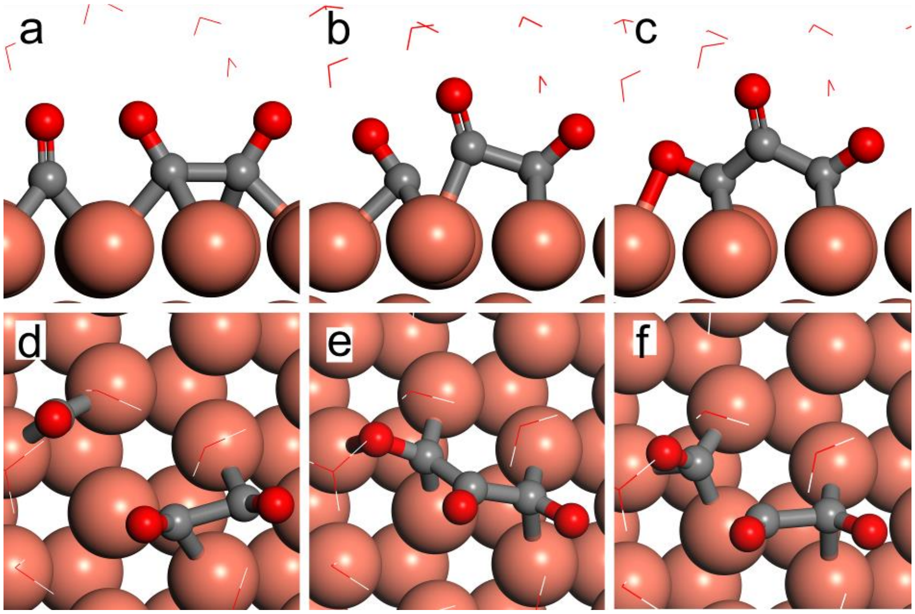

Supplementary Figure 2 | Geometries of C_1 and C_2 coupling on Cu(111) surface. a-c, Side views of initial state (**a**), transition state (**b**), and final state (**c**). **d-f,** Top views of initial state (**d**), transition state (**e**), and final state (**f**).

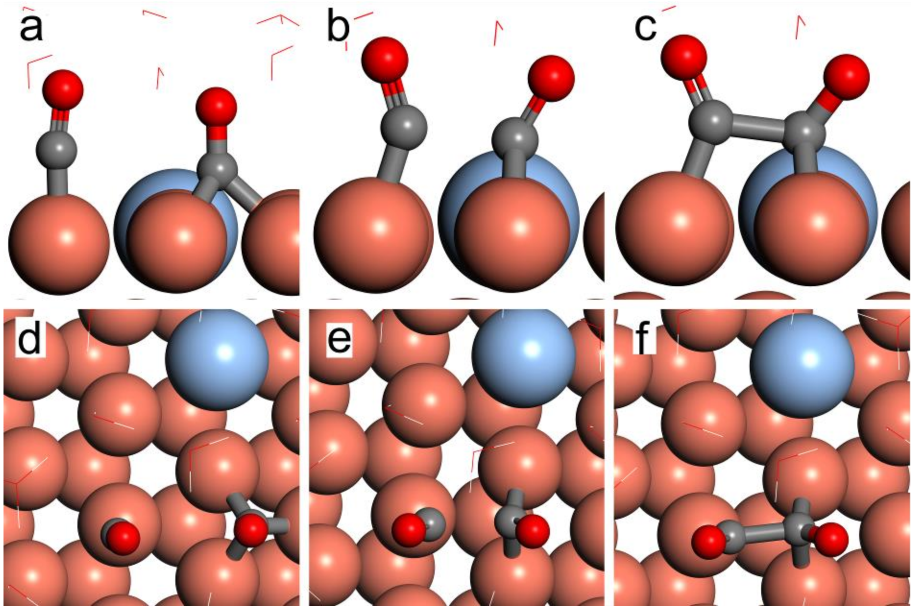

Supplementary Figure 3 | Geometries of CO dimerization on Ag-doped Cu surface. a-c, Side views of initial state (**a**), transition state (**b**), and final state (**c**). **d-f,** Top views of initial state (**d**), transition state (**e**), and final state (**f**). Light blue balls stand for silver atoms. The distance of CO molecules at the transition state of CO dimerization on Ag-doped Cu is 1.908 Å. This notation is used throughout in the Supplementary Information.

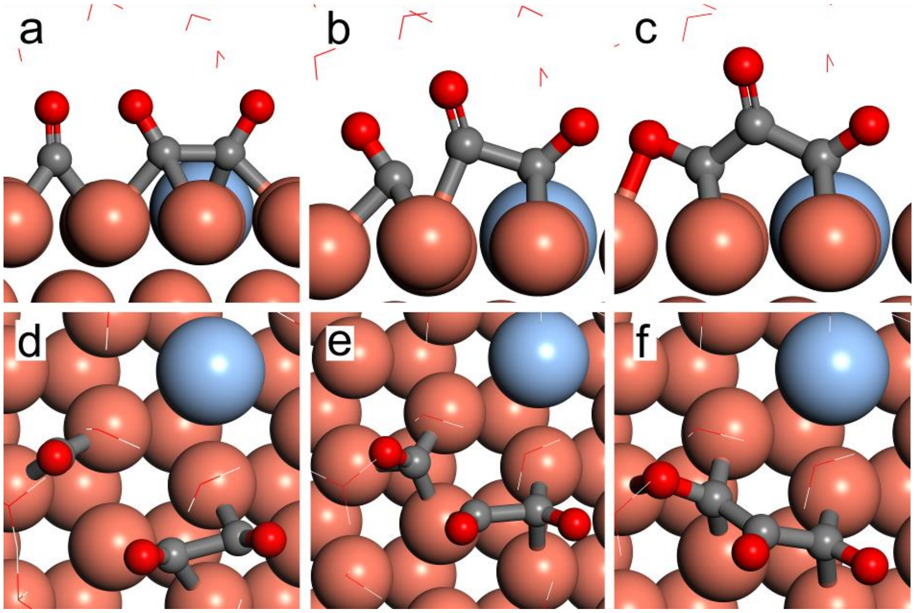

Supplementary Figure 4 | Geometries of C_1 and C_2 coupling on Ag-doped Cu surface. a-c, Side views of initial state (**a**), transition state (**b**), and final state (**c**). **d-f,** Top views of initial state (**d**), transition state (**e**), and final state (**f**). Light blue balls stand for silver atoms.

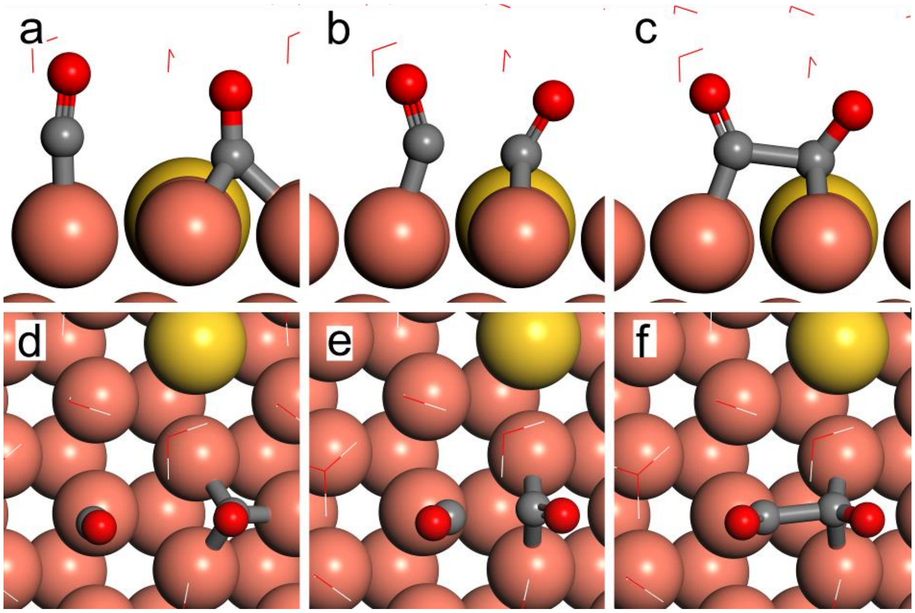

Supplementary Figure 5 | Geometries of CO dimerization on Au-doped Cu surface. a-c, Side views of initial state (**a**), transition state (**b**), and final state (**c**). **d-f,** Top views of initial state (**d**), transition state (**e**), and final state (**f**). Yellow balls stand for gold atoms. This notation is used throughout in the Supplementary Information.

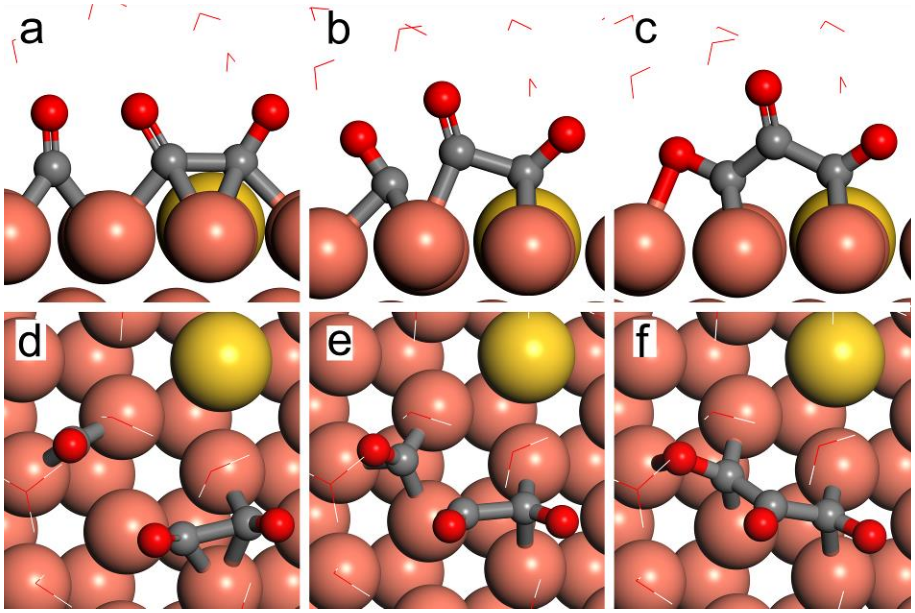

Supplementary Figure 6 | Geometries of C_1 and C_2 coupling on Au-doped Cu surface. a-c, Side views of initial state (**a**), transition state (**b**), and final state (**c**). **d-f,** Top views of initial state (**d**), transition state (**e**), and final state (**f**).

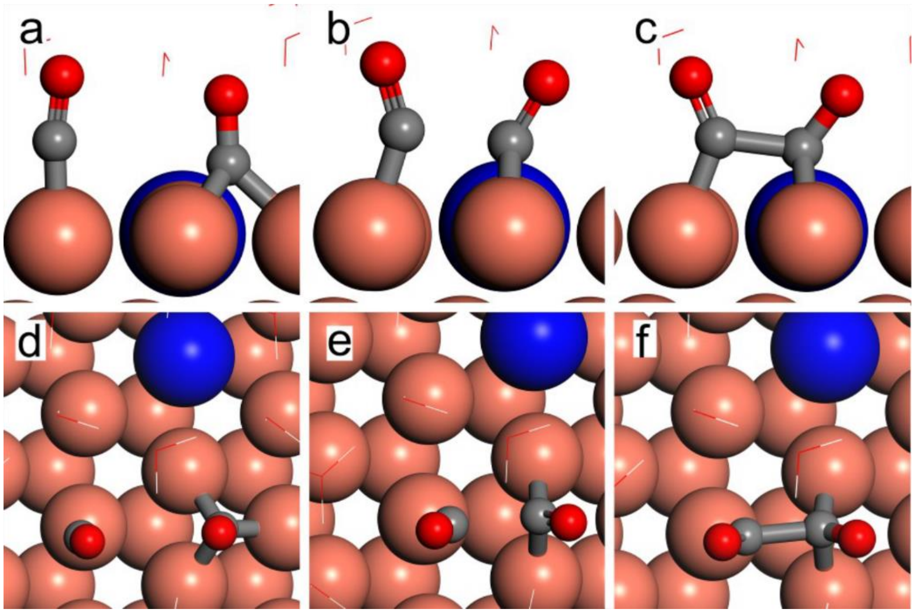

Supplementary Figure 7 | Geometries of CO dimerization on Pd-doped Cu surface. a-c, Side views of initial state (**a**), transition state (**b**), and final state (**c**). **d-f,** Top views of initial state (**d**), transition state (**e**), and final state (**f**). Indigo balls stand for palladium atoms. This notation is used throughout in the Supplementary Information.

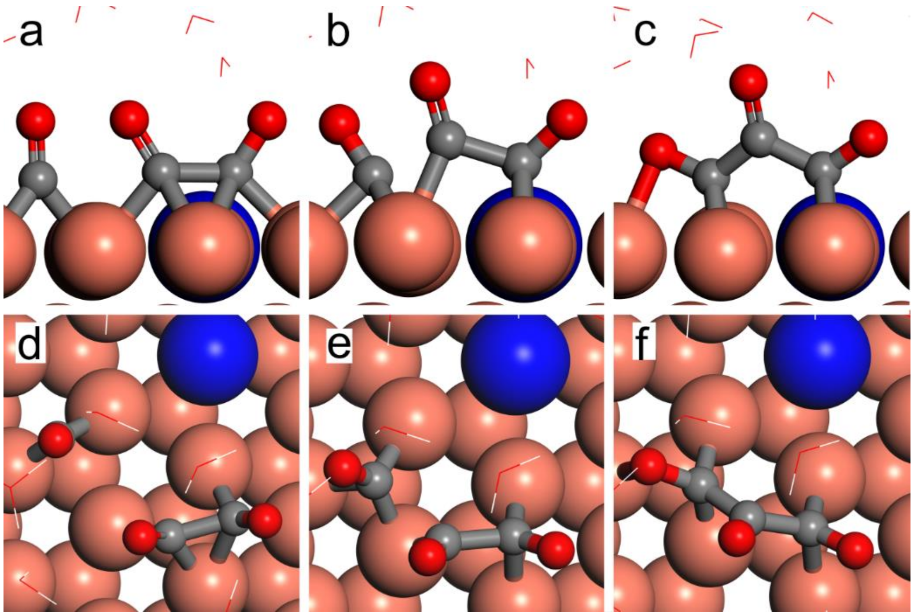

Supplementary Figure 8 | Geometries of C_1 and C_2 coupling on Pd-doped Cu surface. a-c, Side views of initial state (**a**), transition state (**b**), and final state (**c**). **d-f,** Top views of initial state (**d**), transition state (**e**), and final state (**f**).

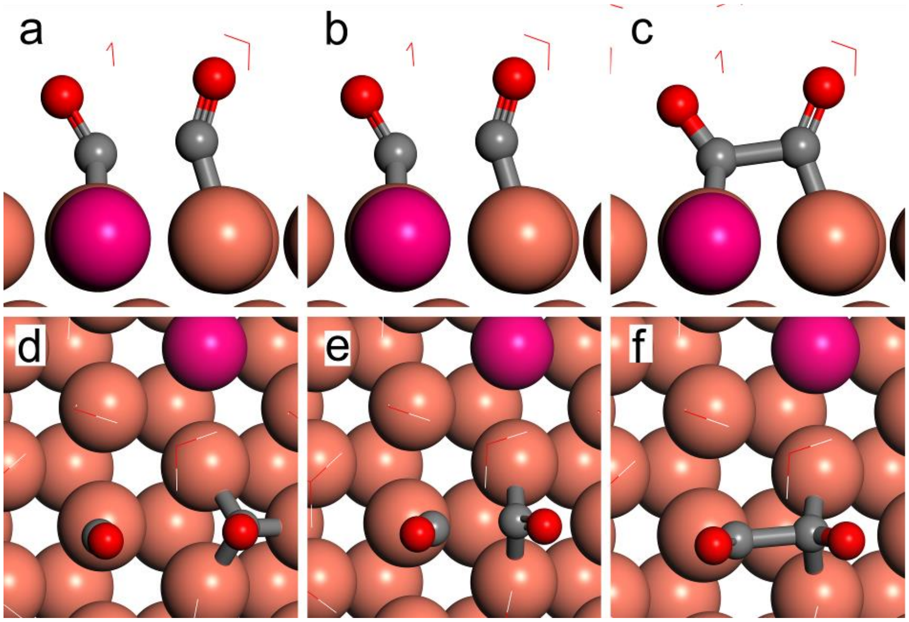

Supplementary Figure 9 | Geometries of CO dimerization on Rh-doped Cu surface. a-c, Side views of initial state (**a**), transition state (**b**), and final state (**c**). **d-f,** Top views of initial state (**d**), transition state (**e**), and final state (**f**). Pink balls stand for rhodium atoms. This notation is used throughout in the Supplementary Information.

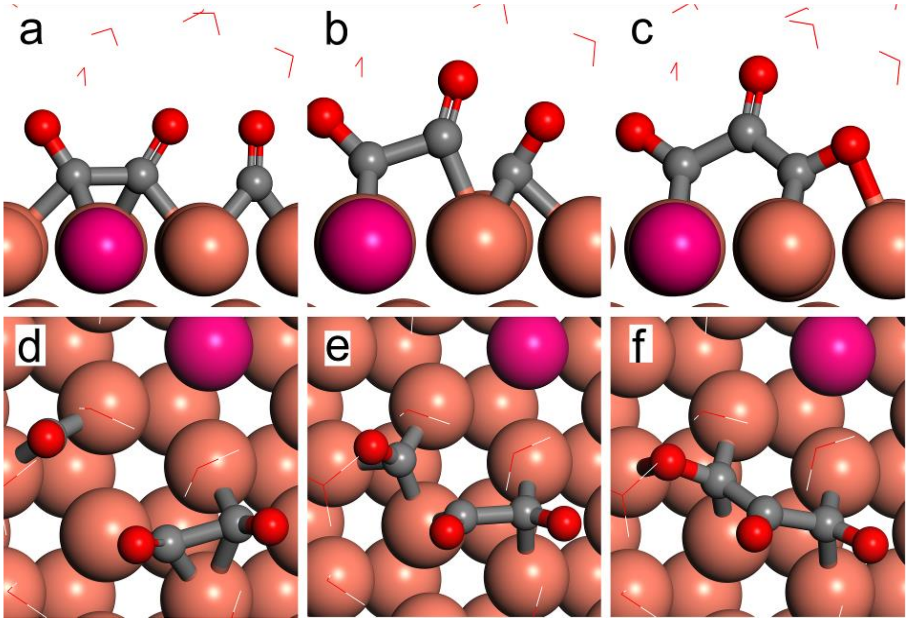

Supplementary Figure 10 | Geometries of C_1 and C_2 coupling on Rh-doped Cu surface. a-c, Side views of initial state (**a**), transition state (**b**), and final state (**c**). **d-f,** Top views of initial state (**d**), transition state (**e**), and final state (**f**).

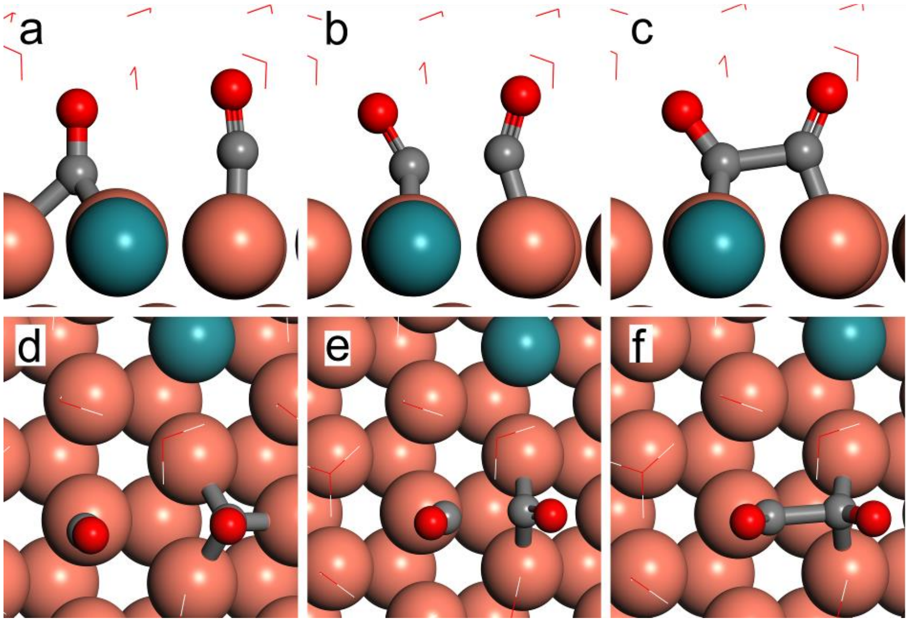

Supplementary Figure 11 | Geometries of CO dimerization on Ru-doped Cu surface. a-c, Side views of initial state (**a**), transition state (**b**), and final state (**c**). **d-f,** Top views of initial state (**d**), transition state (**e**), and final state (**f**). Dark green balls stand for ruthenium atoms. This notation is used throughout in the Supplementary Information.

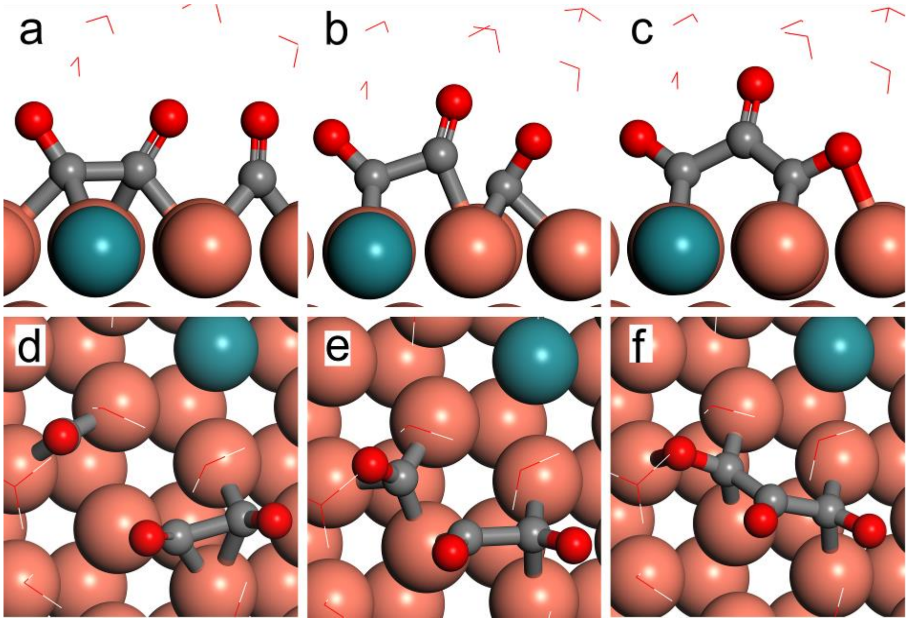

Supplementary Figure 12 | Geometries of C_1 and C_2 coupling on Ru-doped Cu surface. a-c, Side views of initial state (**a**), transition state (**b**), and final state (**c**). **d-f,** Top views of initial state (**d**), transition state (**e**), and final state (**f**).

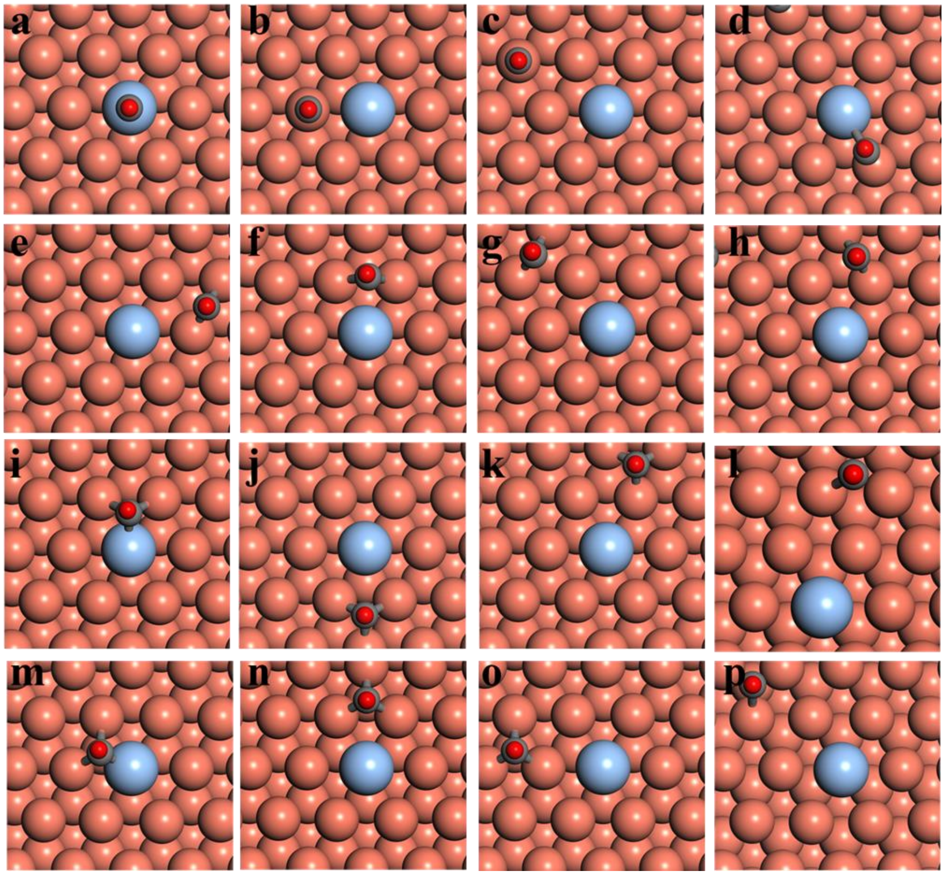

Supplementary Figure 13 | The initial adsorption sites of CO on Ag-doped Cu surface. a, Top site of Ag. **b**, Top site of Cu-b. **c**, Top site of Cu-a. **d**, Bridge site between Ag and Cu-b. **e**, Bridge site between Cu-b and Cu-a. **f**, Bridge site between Cu-b and Cu-b. **g**, Bridge site between Cu-a and Cu-a. **h**, Bridge site between Cu-a and Cu-b. **i**, fcc site of Ag, Cu-b, and Cu-b. **j**, fcc site of Cu-b, Cu-b, and Cu-a. **k**, fcc site of Cu-b, Cu-a, and Cu-a. **l**, fcc site of Cu-a, Cu-a, and Cu-a. **m**, hcp site of Ag, Cu-b, and Cu-b. **n**, hcp site of Cu-b, Cu-b, and Cu-a. **o**, hcp site of Cu-a, Cu-a, and Cu-b. **p**, hcp of Cu-a, Cu-a, and Cu-a. Some optimized adsorption sites are different from initial models, which has been listed in Supplementary Table 2.

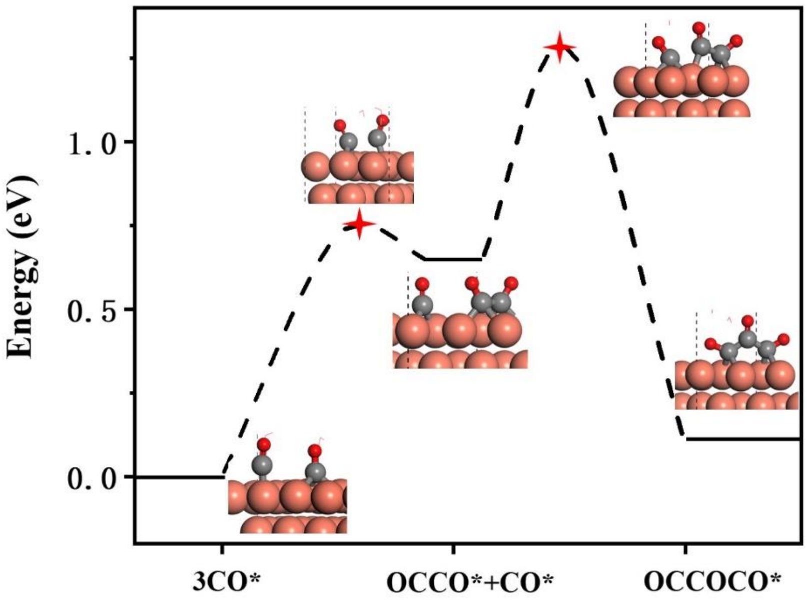

Supplementary Figure 14 | The energy profile of C_1-C_1 coupling and C_1-C_2 coupling on Cu(111). High CO coverage, and the reaction barrier of the carbon-carbon coupling, can each be discovered as distinct points. The high CO coverage is caused by the high CO concentration near the catalyst surface when we utilize the flow cell setup. According to the Langmuir adsorption model, the CO coverage should not affect the carbon-carbon coupling barrier, which is a property of the catalyst. Based on the calculation, the reason why C_1-C_2 coupling barrier is lower than C_1-C_1 coupling is that the intermediate OCCO is less stable compared to CO, and thus it is more active for further reactions. Here we point out, considering many possibilities of C_1-C_2 coupling, the model that reflects the real electrochemical process still needs to be investigated further.

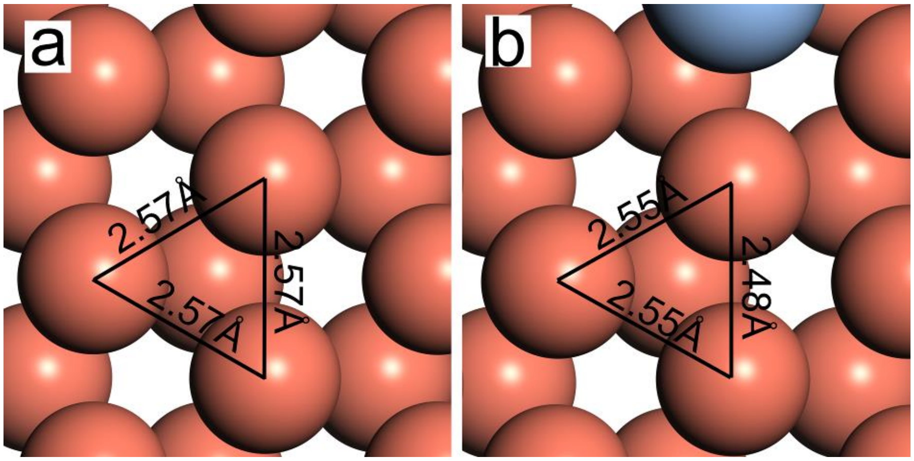

Supplementary Figure 15 | Bond length change due to strain effect on Ag-doped Cu surface.

a,b, Surface geometries with labeled Cu-Cu bond length at CO dimerization active sites on Cu(111) surface (**a**) and Ag-doped Cu surface (**b**).

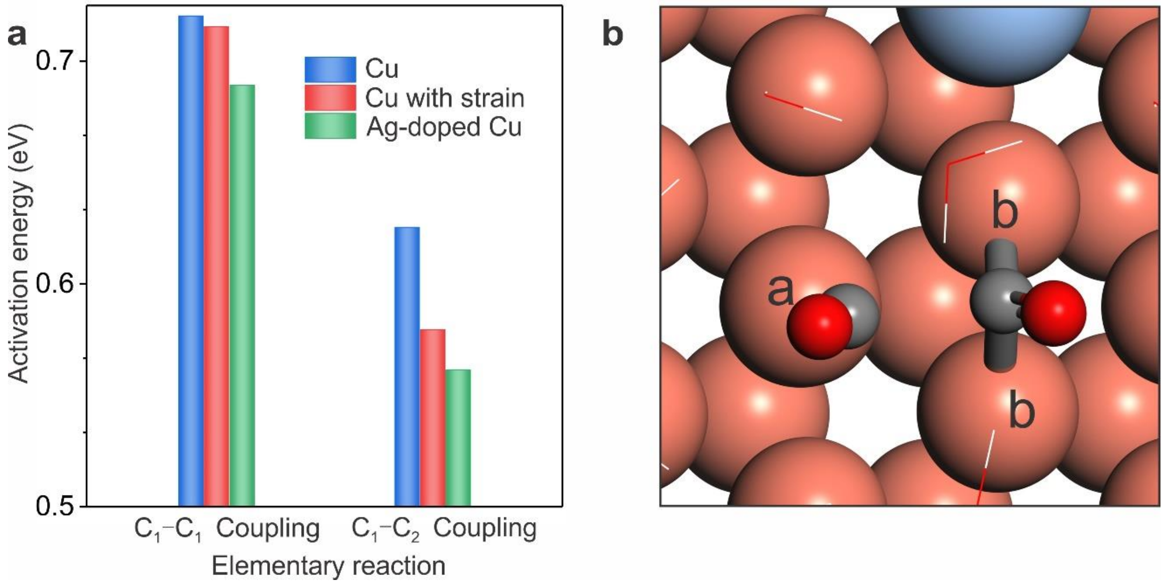

Supplementary Figure 16 | DFT calculations on C_1-C_1 and C_1-C_2 coupling. a, Activation energies of C_1-C_1 and C_1-C_2 coupling on Cu, Cu with strain, and Ag-doped Cu. Cu with strain is the Cu surface with the same bond length as Ag-doped Cu but without Ag substitution. **b,** Two types of neighbouring Cu atoms labeled as a and b on Ag-doped Cu surface.

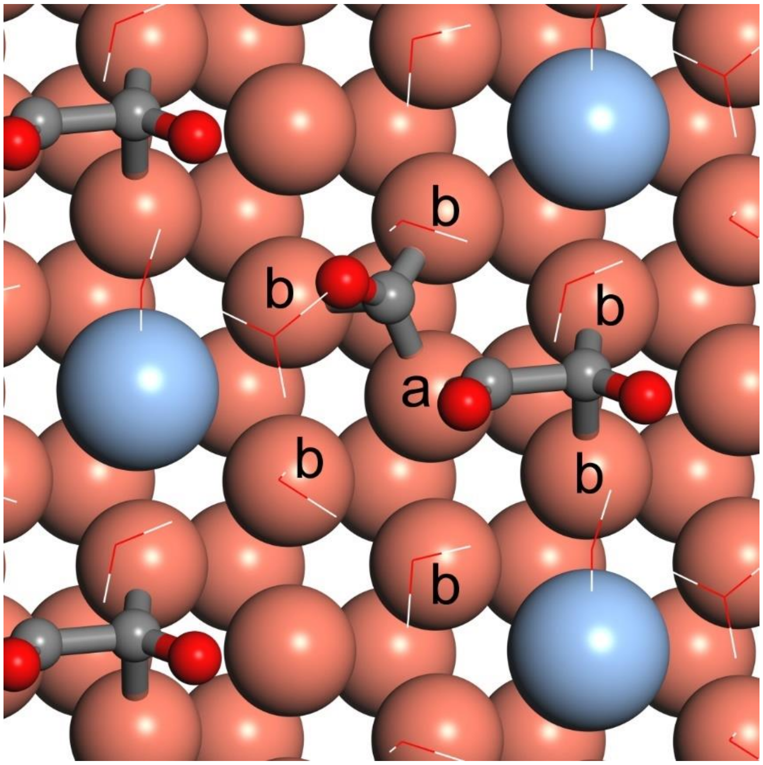

Supplementary Figure 17 | Geometries of C_1 and C_2 coupling on Ag-doped Cu surface. Cu-a and Cu-b are two different types of surface atoms caused by Ag doping. Cu-a atom coordinates with nine Cu atoms, while Cu-b atom coordinates with eight Cu atoms and one Ag atom.

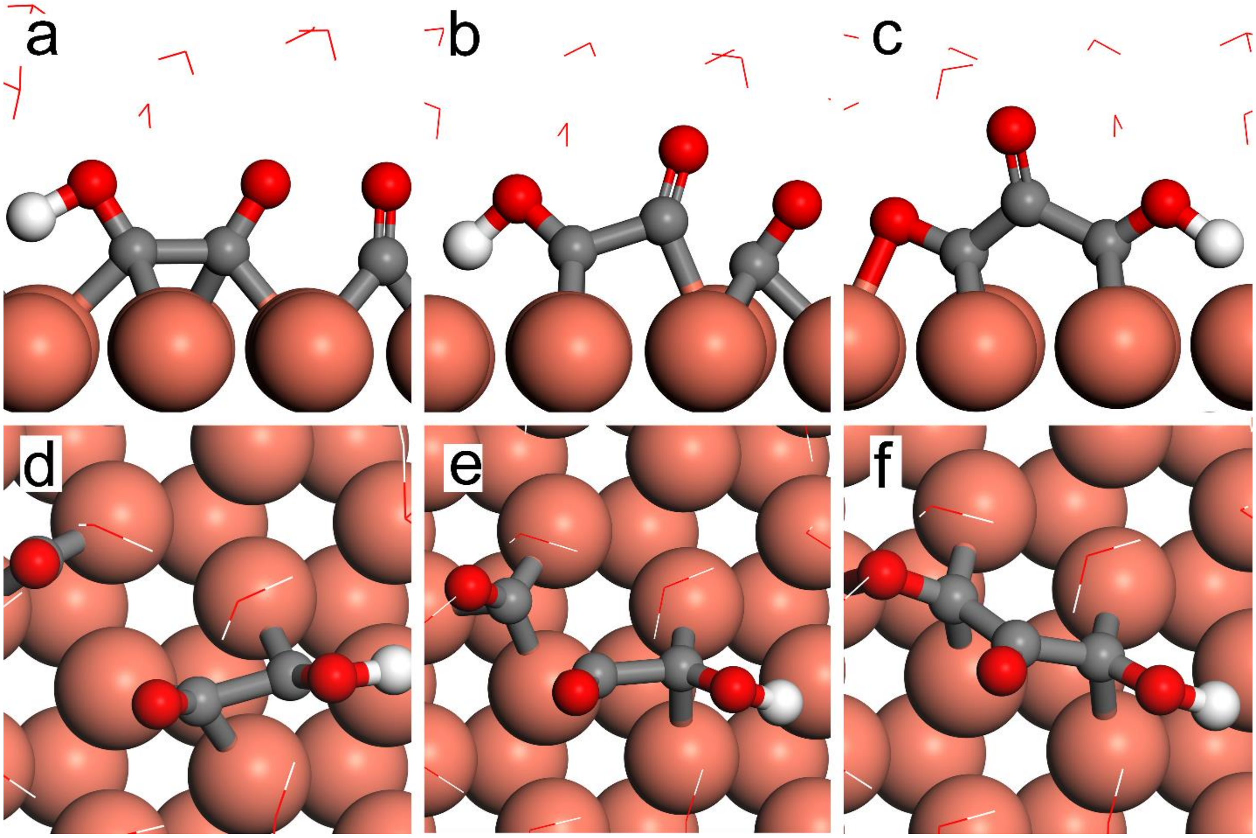

Supplementary Figure 18 | Geometries of CO and OCCOH coupling on Cu surface. a-c, Side views of initial state (**a**), transition state (**b**), and final state (**c**). **d-f,** Top views of initial state (**d**), transition state (**e**), and final state (**f**).

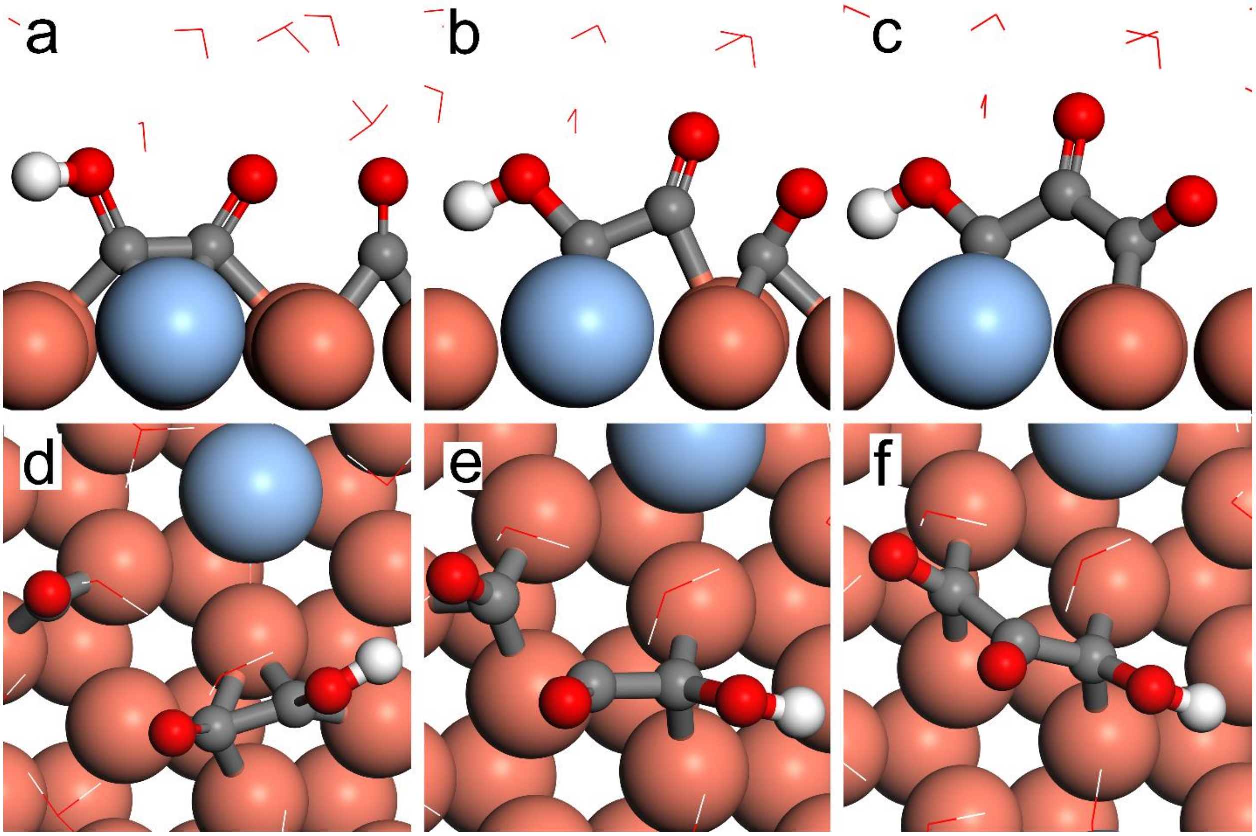

Supplementary Figure 19 | Geometries of CO and OCCOH coupling on Ag-doped Cu surface. **a-c,** Side views of initial state (**a**), transition state (**b**), and final state (**c**). **d-f,** Top views of initial state (**d**), transition state (**e**), and final state (**f**).

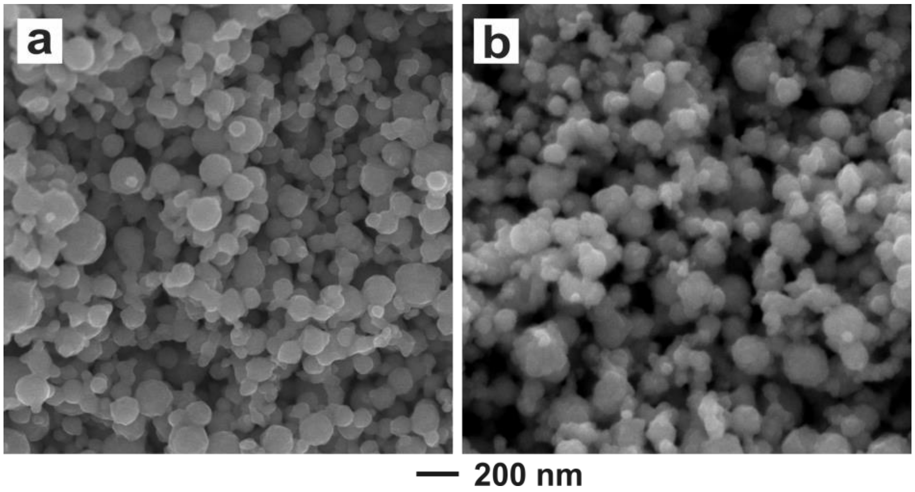

Supplementary Figure 20 | SEM images of catalysts. a, Pristine Cu catalyst. **b,** Ag-doped Cu catalyst.

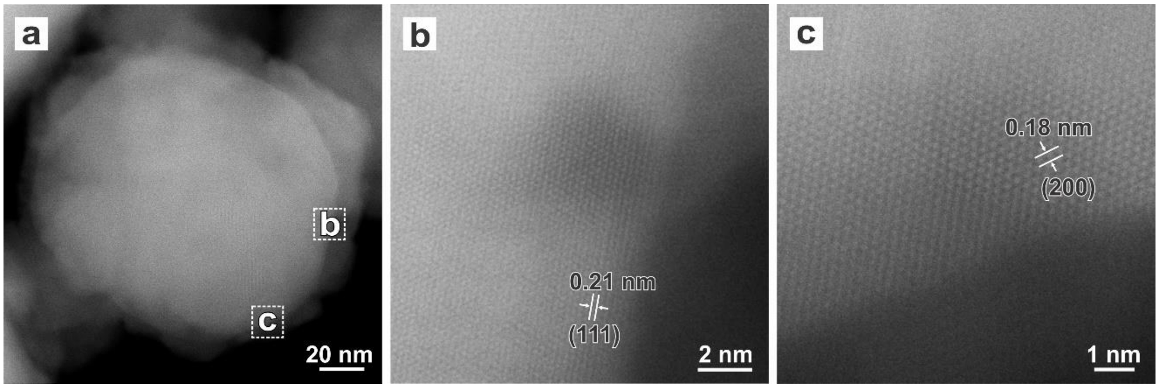

Supplementary Figure 21 | Structural analysis of an Ag-doped Cu nanoparticle. a, HAADF-STEM image of the same Ag-doped Cu nanoparticle in Fig. 2c. **b,c,** Atomic-resolution HAADF-STEM images taken from the edges of a nanoparticle marked by a box in (**a**). The analysis of atomic-resolution HAADF-STEM images demonstrated the both (111) and (100) facets were exposed on Ag-doped Cu nanoparticle.

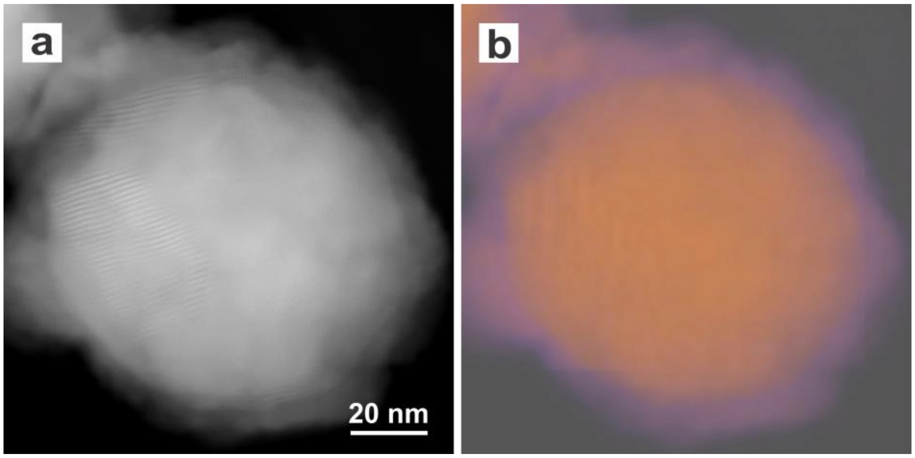

Supplementary Figure 22 | Compositional analysis of an Ag-doped Cu nanoparticle. a, HAADF-STEM image of the same Ag-doped Cu nanoparticle in Fig. 2c. **b,** Overlap of the corresponding EELS elemental mappings of Cu, Ag, and O in Fig. 2c.

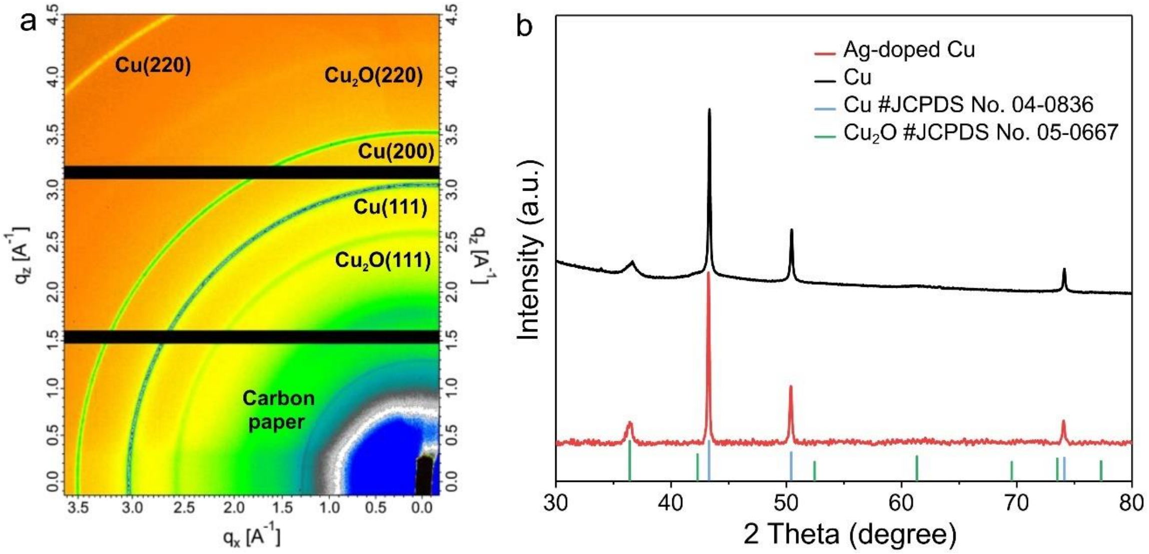

Supplementary Figure 23 | Structural characterizations of GDE. a, WAXS map of Cu GDE. **b,** XRD patterns for Cu and Ag-doped Cu GDE.

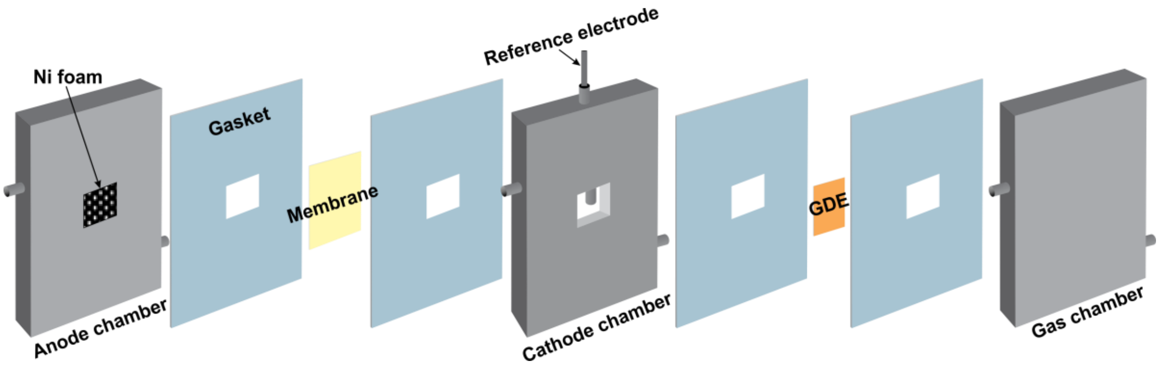

Supplementary Figure 24 | Schematic diagram of designed flow cell reactor. In the configuration of the flow cell reactor, the Ni foam is positioned in the anode chamber. During the electrochemical measurements, the Ni foam is immersed in the anolyte as the OER catalyst. The oxygen bubbles are removed from the cell via the flow of electrolyte.

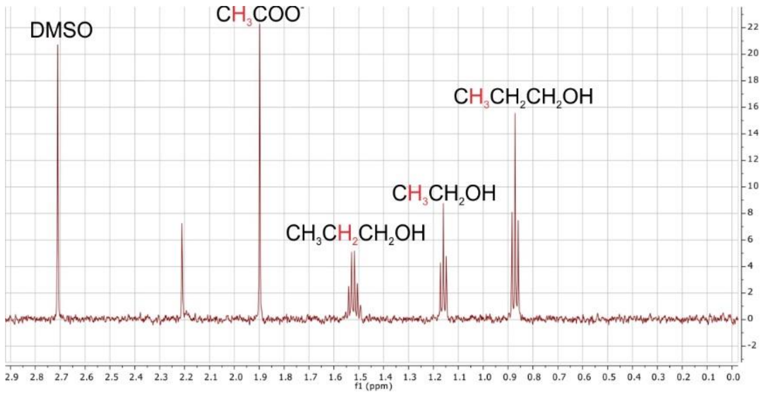

Supplementary Figure 25 | NMR spectrum of liquid products. Representative ^{1}H-NMR spectrum of catholyte after CORR on Ag-doped Cu GDE at -0.46 V_{RHE} in 1M KOH. DMSO is used as an internal standard. The peak near 2.21 ppm is assigned to acetone which is used to wash NMR tubes.

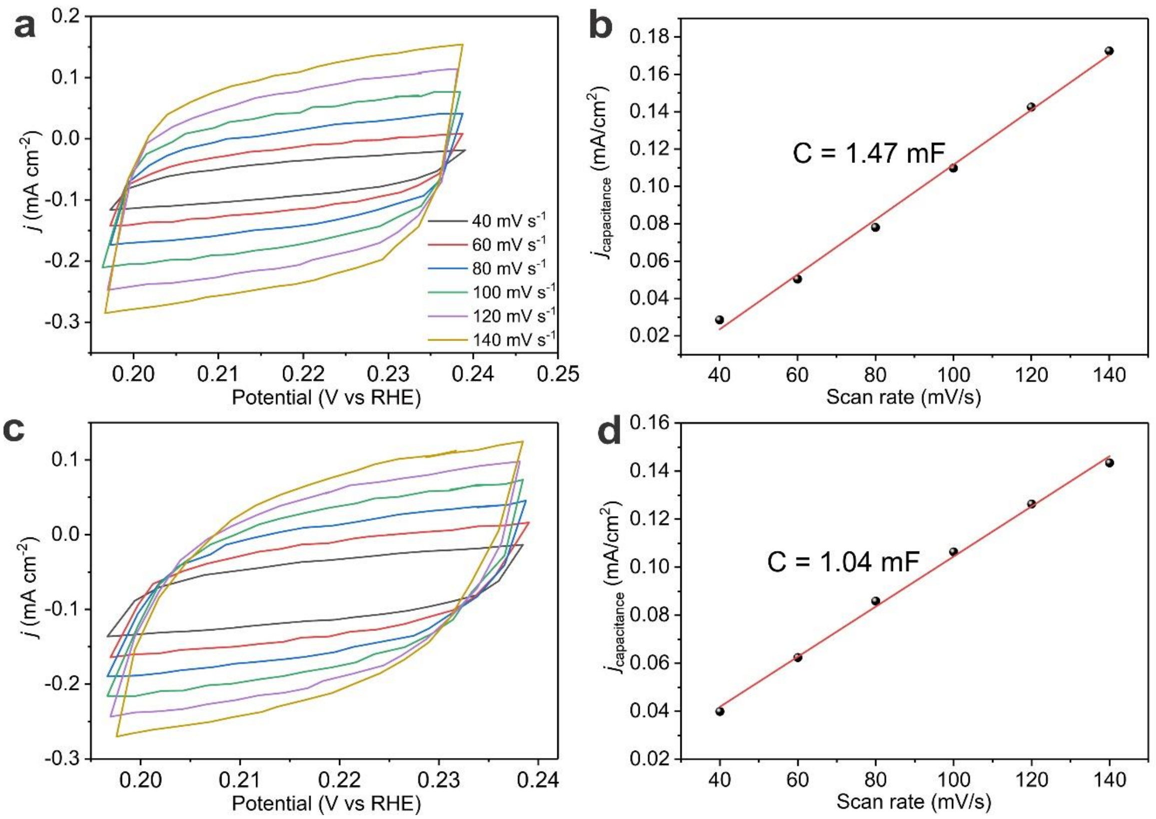

Supplementary Figure 26 | Electrochemical surface area measurement. Determination of double-layer capacitances over a range of scan rates for different catalysts in 1 M KOH saturated with Ar: **a**, **b**, Cu catalyst; **c**, **d**, Ag-doped Cu catalyst. The colour scheme in (**a**) also applied to (**c**).

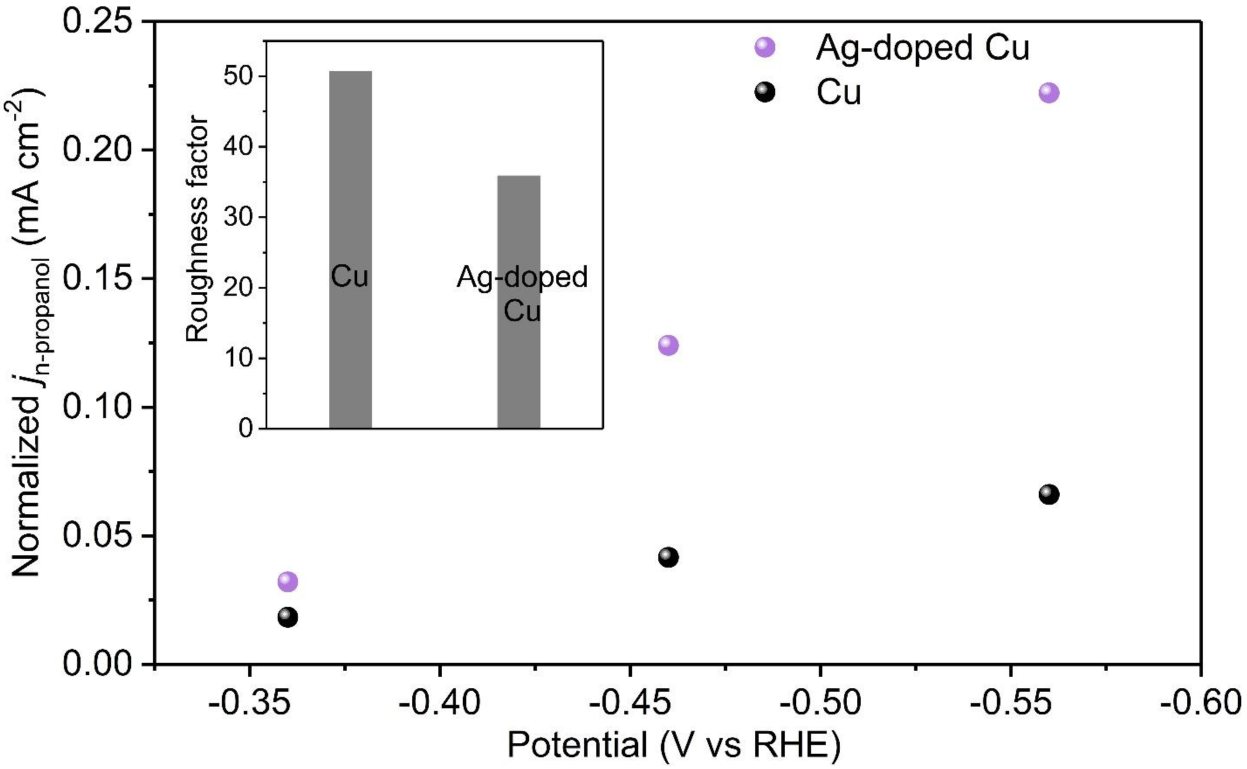

Supplementary Figure 27 | ECSA-normalized partial n-propanol current densities of Ag-doped Cu and Cu catalysts. Inset, surface roughness factors of catalysts calculated by defining the surface roughness factor for electropolished polycrystalline Cu with an electric double layer capacitance of 29 μF as 1 (ref. 14). The potentials shown here are without *i*R compensation.

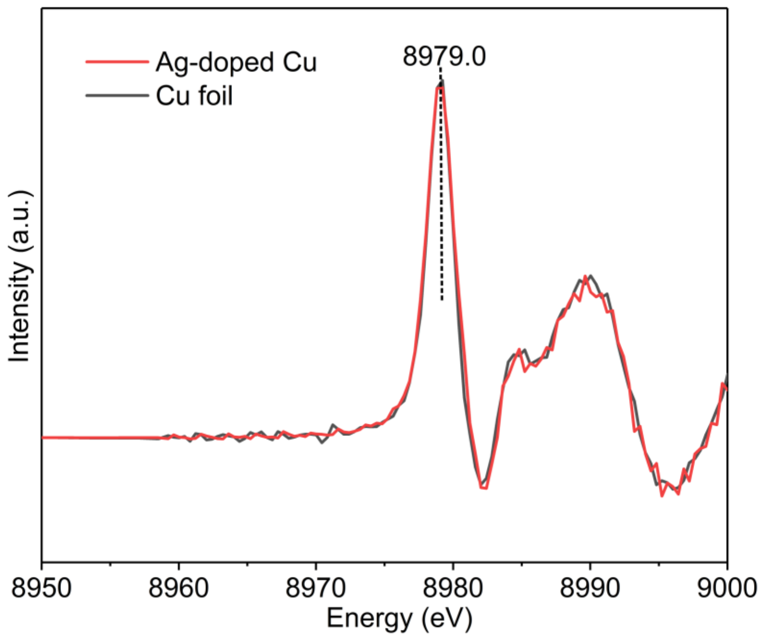

Supplementary Figure 28 | The first derivatives of the Cu K-edge XANES spectra of Ag-doped Cu following 10 s at -0.46 V_{RHE} during CORR.

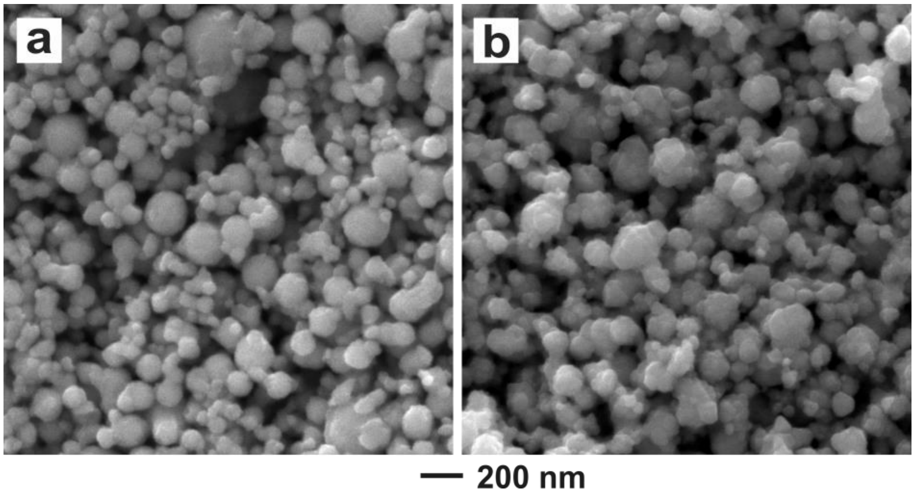

Supplementary Figure 29 | SEM images of catalysts. a, Cu-Ag-20 min catalyst. **b,** Cu-Ag-2 h catalyst.

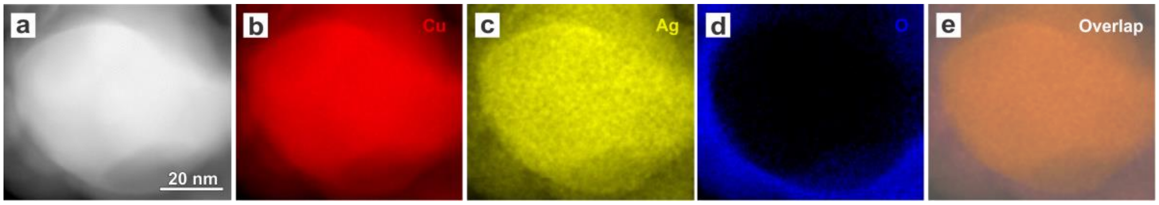

Supplementary Figure 30 | Compositional characterization of a Cu-Ag-20 min nanoparticle. **a,** HAADF-STEM image of a Cu-Ag-20 min nanoparticle. **b-d,** The corresponding EELS elemental mappings of Cu, Ag, and O. In Supplementary Fig. 30d, little oxygen signal can be detected in the center of nanoparticle, suggesting that the concentration of Cu oxide in this part is very low. **e**, Overlap of the corresponding EELS elemental mappings of Cu, Ag, and O.

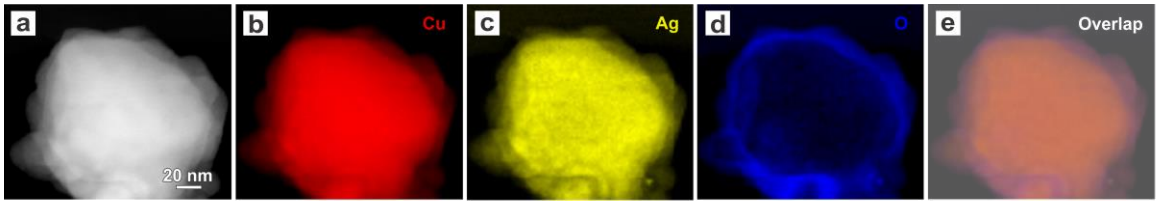

Supplementary Figure 31 | Compositional characterization of a Cu-Ag-2 h nanoparticle. a, HAADF-STEM image of a Cu-Ag-2 h nanoparticle. **b-d,** The corresponding EELS elemental mappings of Cu, Ag, and O. **e**, Overlap of the corresponding EELS elemental mappings of Cu, Ag, and O.

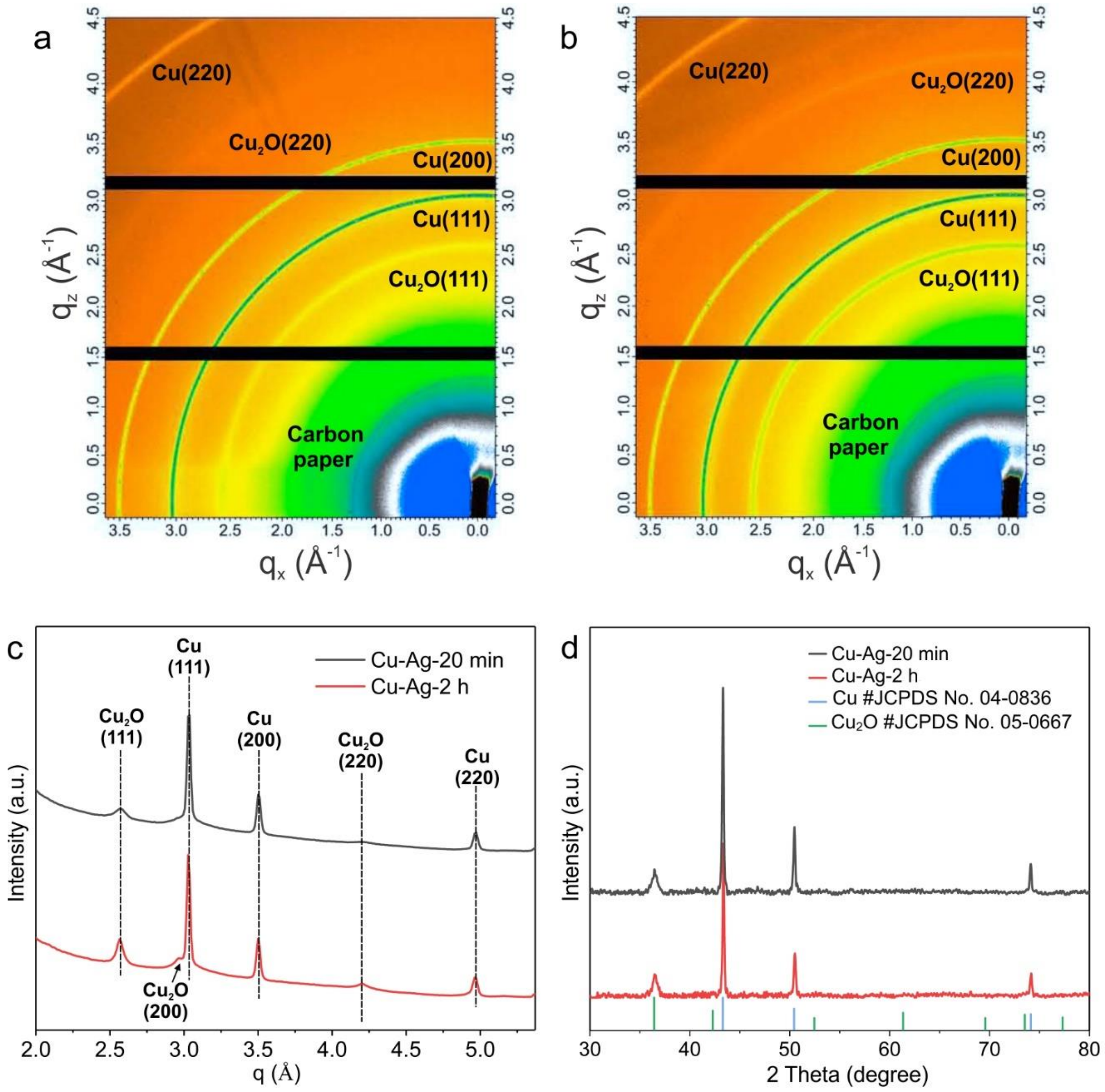

Supplementary Figure 32 | Structural characterizations of GDE. a,b, WAXS maps for Cu-Ag-20 min GDE (**a**) and Cu-Ag-2 h GDE (**b**). **c,** Sector-averages of WAXS maps for Cu-Ag-20 min and Cu-Ag-2 h GDE in Supplementary Fig. 31a and b. **d,** XRD patterns for Cu-Ag-20 min and Cu-Ag-2 h GDE.

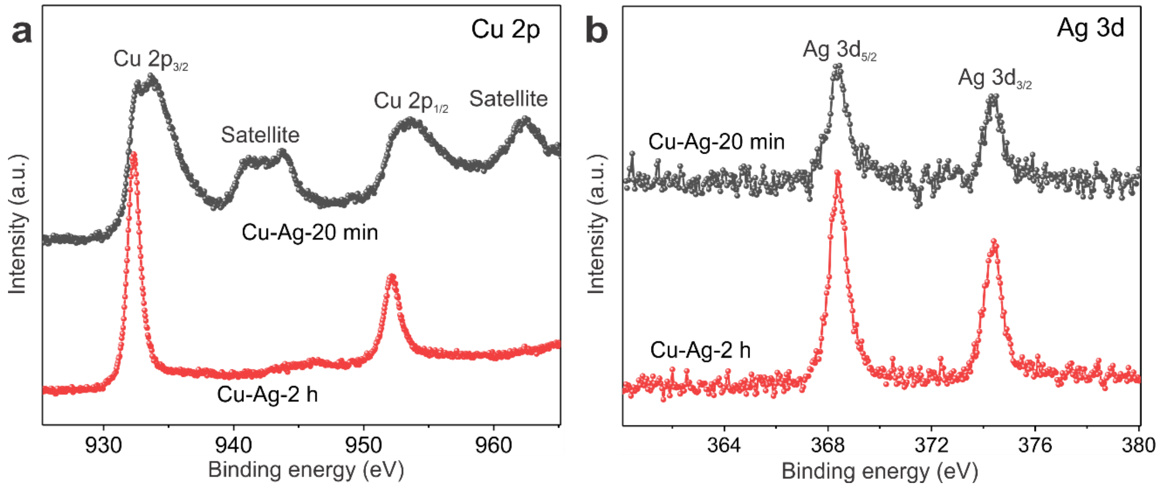

Supplementary Figure 33 | XPS spectra of Cu-Ag-20 min and Cu-Ag-2 h GDE. a, High-resolution Cu 2p spectra of Cu-Ag-20 min and Cu-Ag-2 h GDE. **b,** High-resolution Ag 3d spectra of Cu-Ag-20 min and Cu-Ag-2 h GDE, demonstrating that the valence of silver in the two samples is 0.

Based on the WAXS and XRD results in Supplementary Fig. 32, both Cu-Ag-20 min and Cu-Ag-2 h GDE contained Cu_2O due to the oxidation of Cu during preparation. Cu 2p XPS results showed that Cu-Ag-20 min GDE contained CuO on the surface, while no CuO could be observed in Cu-Ag-2 h GDE. These results suggest that the increase of Ag concentration in the samples can suppress the further oxidation of Cu_2O to CuO.

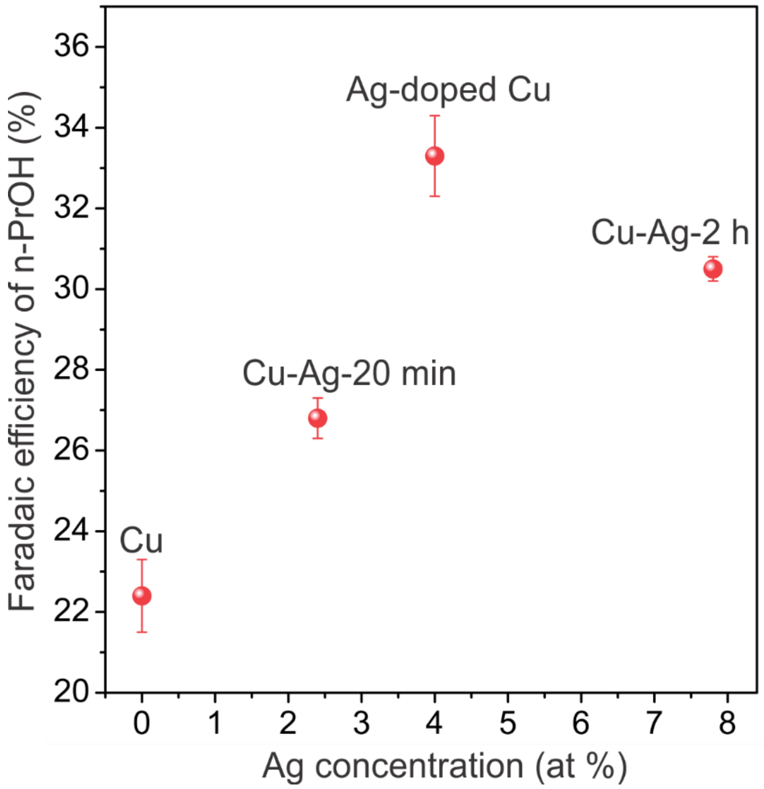

Supplementary Figure 34 | FEs of n-propanol on Ag-doped Cu with different atomic percentages (at %) of Ag and pristine Cu under CORR at a constant potential of -0.46 V_{RHE}.

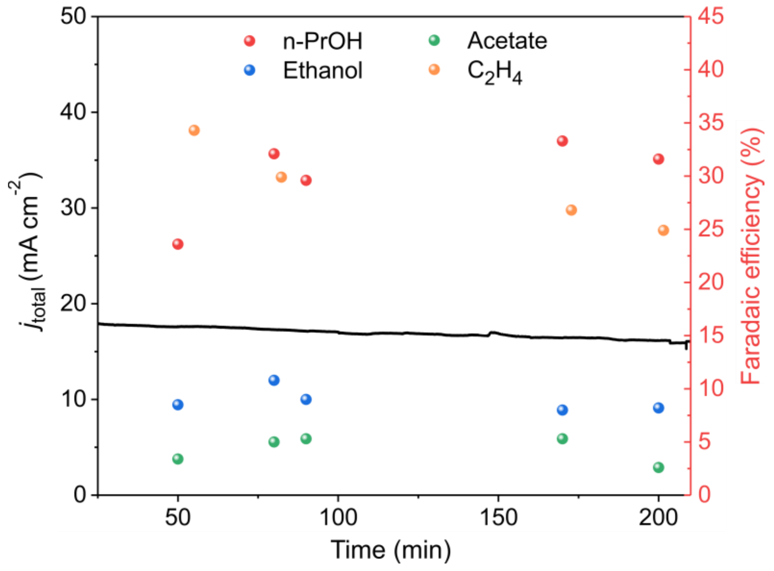

Supplementary Figure 35 | FEs of n-propanol, ethanol, acetate, and ethylene on Ag-doped Cu PTFE electrode during the operation of CORR for 200 min.

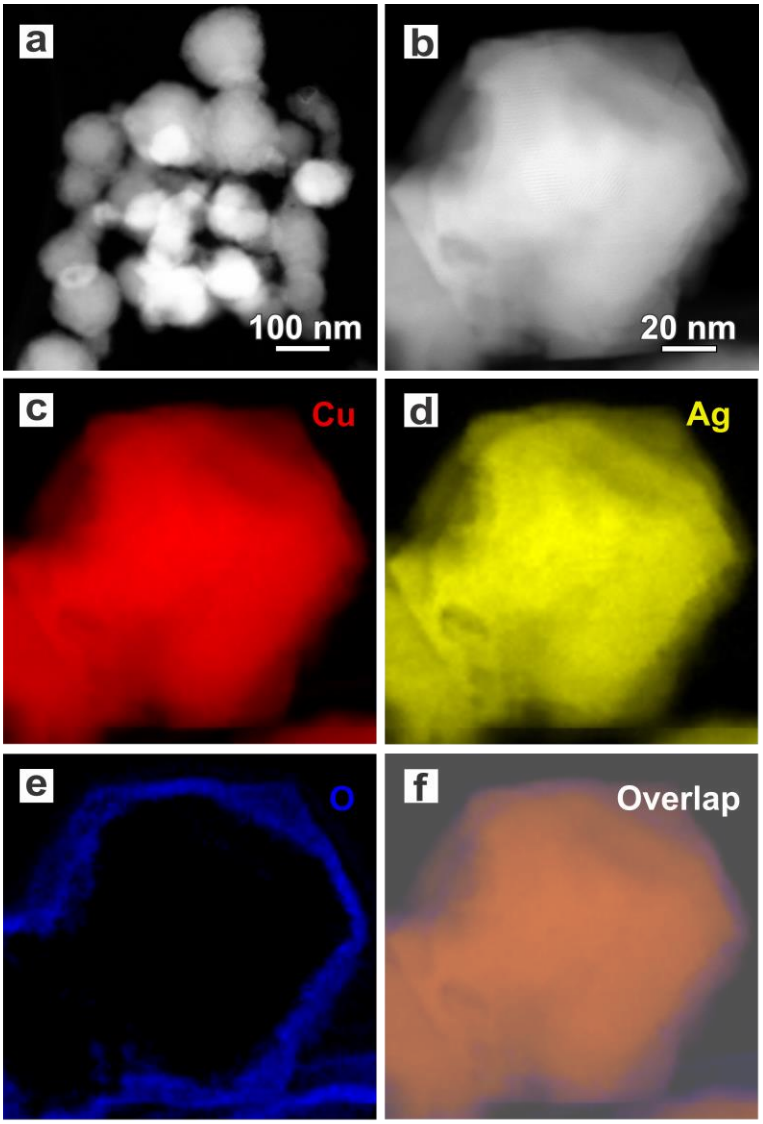

Supplementary Figure 36 | Structural and compositional analyses of Ag-doped Cu nanoparticles after running CORR for 200 min. a, Low magnification HAADF-STEM image of Ag-doped Cu nanoparticles. **b,** HAADF-STEM image of an Ag-doped Cu nanoparticle. **c-e,** The corresponding EELS elemental mappings of Cu, Ag, and O. **f**, Overlap of the corresponding EELS elemental mappings of Cu, Ag, and O. After the reaction, nanocatalysts were oxidized again during the preparation of the TEM sample in air. Therefore, oxygen was detected in EELS elemental mapping.

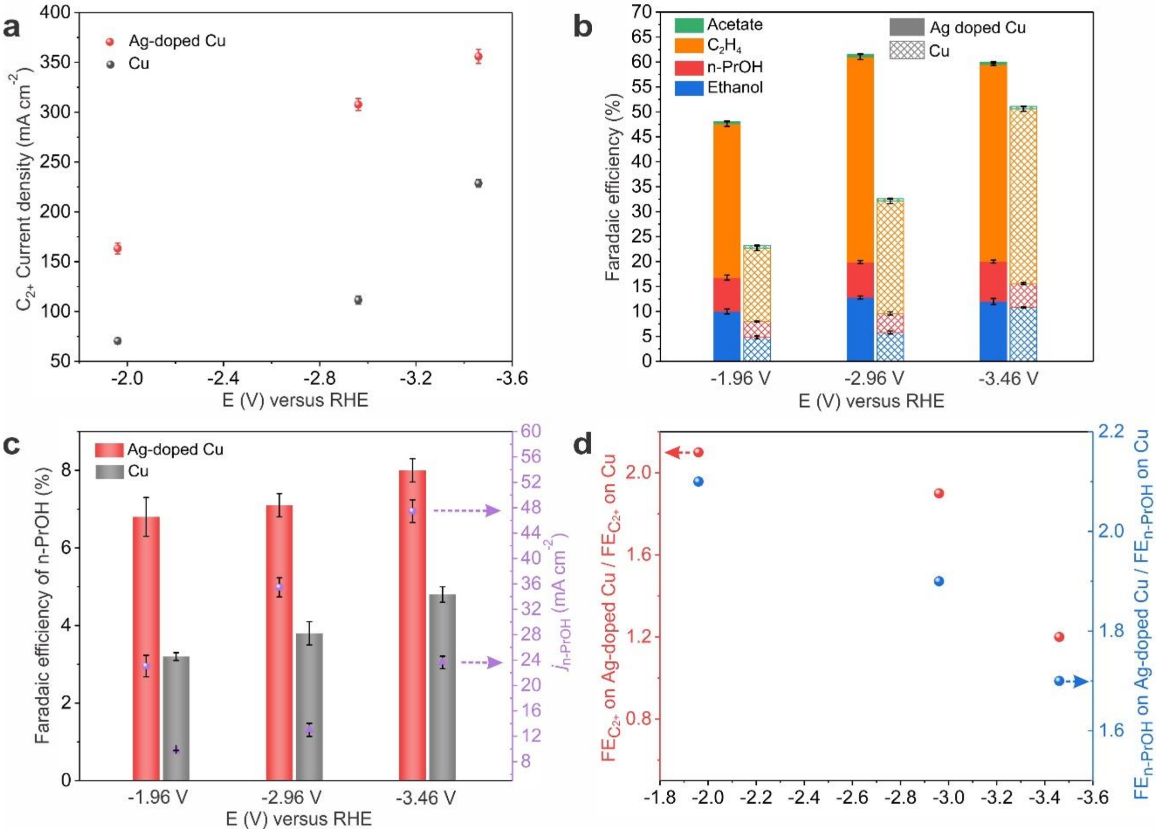

Supplementary Figure 37 | CO_2 electroreduction performance of Ag-doped Cu and Cu catalyst in flow cell. a, Partial current density of total C_{2+} products on Ag-doped Cu and pristine Cu under different potentials. **b**, FEs of acetate, ethylene, n-propanol, and ethanol on Ag-doped Cu and pristine Cu catalysts under different potentials. **c**, Comparison of FEs and partial current density of n-propanol on Ag-doped Cu and Cu catalysts under different potentials. **d**, Comparison of C_{2+} and n-propanol FEs on Ag-doped Cu and Cu catalysts. All the potentials shown here are without *i*R compensation.

Supplementary Table 1. Reaction barriers of C_1-C_1 and C_1-C_2 coupling on Cu and M-doped Cu surfaces.

	E_a (C_1-C_1) (eV)	E_a (C_1-C_2) (eV)
Cu	0.72	0.65
Ag-doped Cu	0.69	0.61
Au-doped Cu	0.70	0.64
Pd-doped Cu	0.80	0.75
Ru-doped Cu	0.74	0.61
Rh-doped Cu	0.79	0.62

Supplementary Table 2. CO adsorption energies (E_{ad}) on different sites of Ag-doped Cu.

Initial sites	Optimized sites	E_{ad} (eV)	Initial sites	Optimized sites	E_{ad} (eV)
a1	a	-0.37	i	n	-0.97
b	b	-0.84	j	j	-0.96
c	c	-0.82	k	k	-0.96
d	b	-0.80	l	l	-0.96
e	e	-0.92	m	b	-0.84
f	n	-0.97	n	n	-0.97
g	o	-0.96	o	o	-0.96
h	n	-0.97	p	k	-0.96

The sites mentioned in this Table are corresponding to the different sites in Supplementary Fig. 13.

Supplementary Table 3. Activation energies of C_1-C_1 and C_1-C_2 coupling on Cu and Ag-doped Cu with different exchange-correlation functionals.

	E_a (C_1-C_1) /eV		E_a (C_1-C_2) /eV	
	Cu	AgCu	Cu	AgCu
PBE	0.72	0.69	0.65	0.61
BEFF	0.63	0.58	1.36	0.59
PBEsol	0.80	0.62	0.79	0.52
PW91	0.68	0.63	0.68	0.54

Supplementary Table 4. Strain and ligand effect on reaction barriers of C_1-C_1 and C_1-C_2 coupling on Cu, Cu with strain, and Ag-doped Cu.

	E_a (C_1-C_1) (eV)	E_a (C_1-C_2) (eV)
Cu	0.72	0.63
Cu with strain	0.72	0.58
Ag-doped Cu	0.69	0.56

Supplementary Table 5. Reaction barriers and enthalpy changes of CO-OCCCOH on Cu and Ag-doped Cu surfaces.

	E_a (eV)	ΔH (eV)
Cu	0.88	0.05
Ag-doped Cu	0.76	-0.08

At the low applied potential, the calculated barrier of OC-OCCOH on Cu(111) is 0.88 eV ($E_{OC-OCCOH}$), higher than of that of OC-OCCO (0.63 eV, $E_{OC-OCCO}$), indicating that the OC-OCCO is more favorable than OC-OCCOH (Supplementary Table 5).

According to the energetic span[1], the total barriers of OC-OCCO (E_1) and OC-OCCOH (E_2) pathways are:

$$E_1 = \Delta H_{OC-CO} + E_{OC-OCCO} \text{ (equation 1)}$$

$$E_2 = \max\left(\Delta H_{OC-CO} + \Delta H_{OCCOH} + E_{OC-OCCOH} + eU,\ E_{OC-OCCOH}\right) \text{ (equation 2),}$$

where U is the applied potential *vs*. the computational hydrogen electrode (CHE) (ref. 2). The reaction energy of CO dimerization is 0.65 eV (ΔH_{OC-CO}), and the reaction energy of OCCO hydrogenation is -0.05 eV (ΔH_{OCCOH}). Therefore, E_2 decreases with the increase of the applied potential. As the reaction potential showed the highest $FE_{n\text{-propanol}}$ in our experiment is -0.46 V_{RHE} (equal to -1.286 V_{CHE}), the total barrier is 0.88 eV, giving the maximum turnover frequency (TOF) of 0.01 s^{-1}.

The total barrier of OC-OCCOH on Ag-doped Cu is 0.76 eV (Supplementary Fig.19), lower than that on pure Cu, giving a maximum TOF of 0.87 s^{-1} at reaction applied potential (-1.286 V_{CHE}). Therefore, after considering the proton/electron transfer in C_1-C_2 coupling, the designed Ag-doped Cu also favors C_1-C_2 coupling reaction compared to Cu.

Supplementary Table 6. Ag concentrations in different types of Ag-doped Cu GDE determined using XPS.

	Ag-doped Cu	Cu-Ag-20 min	Cu-Ag-2 h
Atomic percentage of Ag (%)	4.0	2.4	7.8

Supplementary Table 7. Product FEs for different nanocatalysts under different applied potentials in 1 M KOH electrolyte in CORR.

Catalysts	Potentials[1] (V_{RHE})	$FE_{n\text{-}propanol}$ (%)	$FE_{ethanol}$ (%)	$FE_{acetate}$ (%)	$FE_{ethylene}$ (%)	$FE_{hydrogen}$ (%)	FE_{total} (%)
Cu	-0.36	20.2±0.3	12.5±1	7.4±1	17.1±1.1	18.0±3	~75.2
	-0.46	22.4±0.9	9.0±1	5.8±0.9	26.5±1	28.3±4	~92
	-0.56	17.1±0.8	5.0±1.1	6.1±0.6	32.2±1	18.2±4	~78.6
Ag-doped Cu	-0.36	23.6±0.5	7.4±0.7	9.6±0.6	18.0±2	25.2±2	~83.8
	-0.46	33.3±1	5.9±2	4.7±0.8	29.7±3	23±3	~96.6
	-0.56	23.8±1.1	8.1±1.9	4.4±0.4	43.9±1.8	20.2±2	~100.4
Cu-Ag-20 min	-0.36	28.6±0.7	16.1±1.2	16.0±1	14.4±0.9	9.0±2	~83.1
	-0.46	26.8±0.5	13.4±1	4.9±1	34.1±2	20.6±3	~99.8
	-0.56	21.7±1.2	6.5±0.4	4.5±0.4	32.4±3	29.5±1	~94.6
Cu-Ag-2 h	-0.36	31.2±0.5	6.1±0.6	8.6±1	15.1±1.1	8.2±2	~69.2
	-0.46	30.5±0.3	8.4±1	7.2±1.3	26.5±1.7	17.1±1.1	~89.7
	-0.56	24.3±1	9.1±0.5	9.4±0.2	34.7±2	12.6±3	~90.1

[1] Potentials shown here are without *i*R compensation.
Error bars in this Table represent the standard deviation based on three separate measurements.

Supplementary Table 8. Comparison of n-propanol production via electroreduction CO_2 or CO on Cu-based catalysts.

Catalysts	Potentials (V_{RHE})	Overpotentials (mV)	N-propanol FEs (%)	**N-propanol $EE_{cathodic\ half-cell}$ (%)**	Reactions	References
**Ag-doped Cu**	**-0.416[a]**	**616**	**33±1**	**20.7±0.6**	**CORR**	**This work**
**Cu-Ag-2 h**	**-0.34[a]**	**540**	**31±0.5**	**20.3±0.3**	**CORR**	**This work**
Oxide-derived Cu	--	--	5.4	--	CO_2RR	*J. Phys. Chem. C* **120**, 20058-20067 (2016) (Ref. 15)
Agglomerated Cu nanocrystals	-0.95	1050	10.6	5.5	CO_2RR	*J. Phys. Chem. Lett.* **7**, 20-24 (2016). (Ref. 16)
Cu nanoparticles	-0.81	910	5.9	3.0	CO_2RR	*Proc. Natl. Acad. Sci. U. S. A.* **114**, 10560-10565 (2017) (Ref. 17)
Activated Cu mesh	-0.9	1000	13.1	6.9	CO_2RR	*ACS Catal.* **7**, 7946-7956 (2017) (Ref. 18)
Metal ion cycled Cu	-0.96	1060	15	7.7	CO_2RR	*Nat. Catal.* **1**, 111-119 (2018) (Ref. 19)
Cu_2S–Cu-V	-0.95	1050	8±0.7	4.9±0.4	CO_2RR	*Nat. Catal.* **1**, 421-428 (2018) (Ref. 20)
Oxide-derived Cu	-0.4	600	10.0	6.3	CORR	*Nature* **508**, 504-507 (2014) (Ref. 14)
Oxide-derived Cu	-0.4	600	8.8	5.6	CORR	*J. Am. Chem. Soc.* **137**, 9808-9811 (2015) (Ref. 21)
Cu nanoparticles	-0.5	700	≈6	3.6	CORR	*ACS Cent. Sci.* **2**, 169-174 (2016) (Ref. 22)
Cu nanowires	-0.45	650	1.8	1.1	CORR	*ACS Catal.* **7**, 4467-4472 (2017) (Ref. 23)
Oxide-derived Cu	-0.42	620	25.6	16.0	CORR	*Nat. Catal.* **1**, 748-755 (2018) (Ref. 24)
Cu adparticles	-0.47	670	23	13.9	CORR	*Nat. Commun.* **9**, 4614 (2018) (Ref. 25)
Cavity Cu	-0.56	760	21±1	12.0±0.6	CORR	*Nat. Catal.* **1**, 946-951 (2018) (Ref. 26)

[a] Potential corrected by ohmic loss.
Error bars in this Table represent the standard deviation based on three separate measurements.

Supplementary Table 9. ECSA-normalized partial n-propanol current densities of Ag-doped Cu catalyst.

Potentials (after *i*R compensation, V_{RHE})	ECSA-normalized $j_{n\text{-}propanol}$ (mA cm^{-2})
-0.344	0.03
-0.416	0.12
-0.449	0.22

Supplementary Table 10. Uncompensated resistances of the electrochemical cells for Ag-doped Cu, Cu-Ag-2h, and Cu GDE measured by EIS.

	Ag-doped Cu	Cu-Ag-2h	Cu
Resistances (Ω)	4.72	4.66	5.33

Supplementary Table 11. EXAFS fitting parameters of Ag-doped Cu catalyst and Cu foil.

Samples	Shells	N	R (Å)	$\sigma^2 \times 10^3$ (Å^2)	ΔE_0 (eV), Cu K-edge
Cu foil	Cu-Cu	12	2.545(1)	8.1(5)	5.2(6)
Ag-doped Cu	Cu-Cu	9.9(3)	2.538(4)	7.1(4)	3.0(9)
	Cu-Ag	0.9(2)	2.629(7)		

N, coordination number; R, bonding distance; σ^2, Debye-Waller factor; ΔE_0, shift in adsorption edge energy.

Supplementary Table 12. Product FEs for Ag-doped Cu and pristine Cu nanocatalysts under different applied potentials in 1 M KOH electrolyte in CO_2RR.

Catalysts	Potentials[1] (V_{RHE})	Potentials[2] (V_{RHE})	$FE_{n\text{-}propanol}$ (%)	$FE_{ethanol}$ (%)	$FE_{acetate}$ (%)	$FE_{ethylene}$ (%)	$FE_{formate}$ (%)	FE_{CO} (%)	$FE_{hydrogen}$ (%)	FE_{total} (%)
Ag-doped Cu	-1.96	-0.84	6.8±0.5	10±0.5	0.5±0.1	30.8±0.5	6.8±0.3	35.2±0.5	13.6±0.2	~103.7
	-2.96	-1.31	7.1±0.3	12.8±0.3	0.6±0.1	41.1±0.5	4.7±0.3	24.5±0.4	12±0.3	~102.8
	-3.46	-1.50	8.0±0.3	12±0.6	0.5±0.1	39.5±0.2	6.5±0.3	25.5±0.5	11.4±0.7	~103.4
Cu	-1.96	-0.82	3.2±0.1	4.8±0.3	0.5±0.1	14.7±0.5	8.9±0.3	60±1	11.7±0.5	~103.8
	-2.96	-1.68	3.8±0.3	5.8±0.3	0.5±0.1	22.5±0.5	7.6±0.5	50.5±0.4	11.5±0.8	~102.2
	-3.46	-1.79	4.8±0.2	10.8±0.1	0.5±0.1	35.0±0.5	3.8±0.1	31±0.5	11.0±0.5	~96.9

[1] Potentials before *i*R compensation.
[2] Potentials after *i*R compensation.
Error bars in this Table represent the standard deviation based on three separate measurements.

Supplementary Methods.

Theoretical Methods. In this work, all the DFT calculations were carried out with a periodic slab model using the Vienna *ab initio* simulation program (VASP)[3-6]. The generalized gradient approximation (GGA) was used with the Perdew-Burke-Ernzerhof (PBE)[7] exchange-correlation functional. The projector-augmented wave (PAW) method[8,9] was utilized to describe the electron-ion interactions, and the cut-off energy for the plane-wave basis set was 450 eV. In order to illustrate the long-range dispersion interactions between the adsorbates and catalysts, we employed the D3 correction method of Grimme et al. (ref. 10). Brillouin zone integration was accomplished using a 3×3×1 Monkhorst-Pack k-point mesh. All the adsorption geometries were optimized using a force-based conjugate gradient algorithm, while transition states (TSs) were located with a constrained minimisation technique[11-13]. At all intermediate and transition states, one charged layer of water molecules was added to the surface to take the combined field and solvation effects into account[12]. In the CO dimerization, there is no proton or electron transfer, thus the computational hydrogen electrode was not used in this work. For the modelling of Cu(111), the crystal structure was optimized, and Cu(111) was modelled with a periodic four-layer p(4×4) model with the 2 lower layers fixed and 2 upper layers relaxed. Cu(111) was chosen because Cu(111) is more stable relative to Cu(100) (ref. 14), and thus improving the activity of Cu(111) for C_3 formation is more significant. In addition, the overall barrier of C_1 to C_3 product on Cu(100) is higher than that on Cu(111), despite the low barrier of C_1-C_1 dimerization on Cu(100).

We started with CO adsorption on Cu(111) surface as shown in Supplementary Fig. 1a and d. The reaction barrier of CO dimerization is calculated to be 0.72 eV, which is similar to the value proposed by Nørskov and co-workers in the same solvent model[15]. The barrier of CO and OCCO coupling is calculated to be 0.63 eV (Supplementary Fig. 2). Given that C_2 FEs are always higher

than C_3 FEs in previous CORR and CO_2RR reported[16-28], C-C coupling is likely more favorable than concerted C-C-C coupling. Thus, rather than concerted C-C-C coupling, sequential formation of C-C bonds was considered as the main reaction mechanism for the C_3 products formation, which is also the most commonly used mechanism in previous reports[28-30]. To screen the possible metals for doping in Cu, we substituted one surface copper atom with Ag, Au, Pd, Rh, and Ru as shown in Supplementary Figs. 3-12. Based on the above surfaces, we calculated the barriers of C_1-C_1 and C_1-C_2 coupling, and the results are shown in Supplementary Table 1.

To support the geometries we proposed, we calculated the CO adsorption energies on all the possible 16 sites of Ag-doped Cu surface including all of fcc hollow, hcp hollow, bridge, or top sites of Cu-a, Cu-b, and Ag (Cu-a and Cu-b determined by their coordination environment), as shown in Supplementary Fig.13 and Supplementary Table 2. By comparing all the CO adsorption energies on different sites, the configuration with CO adsorbing on hcp site of Cu-b, Cu-b, and Cu-a (Supplementary Fig. 13n) is the most stable and thus were used for our calculation. Due to the similarity of adsorption schemes and structures of CO, OCCO, and OCCOCO, we assume the strongest adsorption sites of CO are also the adsorption sites for OCCO and OCCOCO.

Additionally, we also tested other functionals including Bayesian error estimation functional (BEEF)[31], Perdew-Burke-Ernzerhof revised for solids (PBEsol)[32], and Perdew - Wang 91 (PW91)[33] for the activation energies of C_1-C_1 and C_1-C_2 coupling on Cu and Ag-doped Cu (Supplementary Table 3). Each functional predicts an enhancement of C-C coupling of Ag-doped Cu compared to pure Cu.

The effect of strain is evaluated by using the structures of all transition states and intermediate states on Ag-doped Cu surface and Cu with strain surface. The surface atoms are then fixed, and all the transition states and intermediate states are optimized based on the methods above.

Using the energetic span model[34], the rate of C_2 formation should be $r_{C2}{\sim}e^{-E_{C1-C1}/RT}$, and C_3 formation rate should be $r_{C3}{\sim}e^{-(\Delta H_{C1-C1}+E_{C1-C2})/RT}$, where E_{C1-C1} and E_{C1-C2} are the barrier for C_1-C_1 and C_1-C_2 coupling, and ΔH_{C1-C1} is the enthalpy change for C_1-C_1 coupling. As $\Delta H_{C1-C1} + E_{C1-C2} > E_{C1-C1}$ suggested by Supplementary Fig. 14, C_3 formation rate should be slower than C_2 formation rate according to our calculation, in agreement with our experiment results.

Chemicals and Materials.

Commercial Cu nanopowder (99%) and silver nitrate ($AgNO_3$, 99%) were purchased from Sigma-Aldrich. Potassium hydroxide (KOH) and methanol were purchased from Caledon Laboratory Chemicals. Gas diffusion layer (GDL, Freudenberg H14C9), anion exchange membrane (Fumasep FAB-PK-130) were received from Fuel Cell Store. Ni foam (1.6 mm thickness) was purchased from MTI Corporation. All chemicals were used as received. All aqueous solutions were prepared using deionized water with a resistivity of 18.2 M Ω cm^{-1}.

Calculation for Equilibrium Potential.

Equilibrium potentials for the half reactions of CO to n-propanol and CO_2 to n-propanol were calculated based on the values of the standard molar Gibbs energy of formation at 298.15 K (ref. 35). We assumed that gases are at 1 atm and liquids are in the pure form.

$$3\mathrm{CO(g)} + 12\,e^- + 12\mathrm{H}^+ \rightarrow C_3H_7OH(l) + 2H_2O(l) \qquad \Delta G^\circ = -231.5\ \mathrm{kJ\ mol^{-1}}$$

$$3CO_2\mathrm{(g)} + 18\,e^- + 18\mathrm{H}^+ \rightarrow C_3H_7OH(l) + 5H_2O(l) \qquad \Delta G^\circ = -171.2\ \mathrm{kJ\ mol^{-1}}$$

Based on $E^o = \frac{-\Delta G^o}{nF}$ (equation 1), we can get $E^o_{n-propanol-1}$ = 0.20 V versus RHE and $E^o_{n-propanol-2}$ = 0.10 V versus RHE for the reactions of CO to n-propanol and CO_2 to n-propanol, respectively. Herein, n is the number of electrons transferred and F is the Faraday constant.

Calculation for Cathodic Energy Conversion Efficiency.

The OER in anode side is one of main contributors to the energy lost, but here we excluded the effect of the OER and analyzed the cathode performance using cathodic energy conversion efficiency ($EE_{cathodic\ half\text{-}cell}$), where the overpotential of oxygen evolution is assumed to be 0.

The n-propanol $EE_{cathodic\ half\text{-}cell}$ can be calculated as follows[36]:

$$EE_{cathodic\ half-cell} = \frac{(1.23+(-E_{n-propanol}))\times FE_{n-propanol}}{(1.23+(-E))} \text{ (equation 2),}$$

where E is applied potential versus RHE, $FE_{n\text{-}propanol}$ is the measured Faradaic efficiency of n-propanol in percentage, and $E_{n\text{-}propanol}$ = 0.20 V_{RHE} for CORR or $E_{n\text{-}propanol}$ = 0.10 V_{RHE} for CO_2RR. As shown in the equation above, n-propanol $EE_{cathodic\ half\text{-}cell}$ is governed by both FE and overpotential, which are the two important factors in CORR.

For example, at -0.416 V_{RHE} after *i*R compensation, Ag-doped Cu GDE delivered a n-propanol FE of 33% in CORR. Then the n-propanol $EE_{cathodic\ half\text{-}cell}$ for n-propanol can be calculated as follows:

$$EE_{cathodic\ half-cell} = \frac{(1.23+(-0.20))\times 33\%}{(1.23+(-(-0.416))} = 20.7\%$$

Supplementary References

1. Kozuch, S. & Shaik, S. How to conceptualize catalytic cycles? The energetic span model *Acc. Chem. Res.* **44**, 101-110 (2011).

2. Nørskov, J. K. et al. Origin of the overpotential for oxygen reduction at a fuel-cell cathode. *J. Phys. Chem. B* **108**, 17886-17892 (2004).

3. Kresse, G. & Furthmüller, J. Efficient iterative schemes for *ab initio* total-energy calculations using a plane-wave basis set. *Phys. Rev. B* **54**, 11169-11186 (1996).

4. Kresse, G. & Furthmüller, J. Efficiency of *ab-initio* total energy calculations for metals and semiconductors using a plane-wave basis set. *Comp. Mater. Sci.* **6**, 15-50 (1996).

5. Kresse, G. & Hafner, J. *Ab initio* molecular-dynamics simulation of the liquid-metal–amorphous-semiconductor transition in germanium. *Phys. Rev. B* **49**, 14251-14269 (1994).

6. Kresse, G. & Hafner, J. *Ab initio* molecular dynamics for liquid metals. *Phys. Rev. B* **47**, 558-561 (1993).

7. Perdew, J. P., Burke, K. & Ernzerhof, M. Generalized gradient approximation made simple. *Phys. Rev. Lett.* **77**, 3865-3868 (1996).

8. Kresse, G. & Joubert, D. From ultrasoft pseudopotentials to the projector augmented-wave method. *Phys. Rev. B*, **59**, 1758-1775 (1999).

9. Blöchl, P. E. Projector augmented-wave method. *Phys. Rev. B*, **50**, 17953-17979 (1994).

10. Grimme, S., Antony, J., Ehrlich, S. & Krieg, H. A consistent and accurate ab initio parametrization of density functional dispersion correction (DFT-D) for the 94 elements H-Pu. *J. Chem. Phys.* **132**, 154104 (2010).

11. Michaelides, A. et al. Identification of general linear relationships between activation energies and enthalpy changes for dissociation reactions at surfaces. *J. Am. Chem. Soc.* **125**, 3704-

3705 (2003).

12. Liu, Z.-P. & Hu, P. General rules for predicting where a catalytic reaction should occur on metal surfaces: a density functional theory study of C−H and C−O bond breaking/making on flat, stepped, and kinked metal surfaces. *J. Am. Chem. Soc.* **125**, 1958-1967 (2003).

13. Alavi, A., Hu, P., Deutsch, T., Silvestrelli, P. L. & Hutter, J. CO oxidation on Pt(111): an *ab initio* density functional theory study. *Phys. Rev. Lett.* **80**, 3650-3653 (1998).

14. Wang, Z., Liu, X.; Rooney, D. W. & Hu, P. Elucidating the mechanism and active site of the cyclohexanol dehydrogenation on copper-based catalysts: A density functional theory study. *Surf. Sci.*, **640**, 181-189 (2015).

15. Montoya, J. H., Shi, C., Chan, K. & Nørskov, J. K. Theoretical insights into a CO dimerization mechanism in CO_2 electroreduction. *J. Phys. Chem. Lett.* **6**, 2032-2037 (2015).

16. Li, C. W., Ciston, J. & Kanan, M. W. Electroreduction of carbon monoxide to liquid fuel on oxide-derived nanocrystalline copper. *Nature* **508**, 504-507 (2014).

17. Handoko, A. D. et al. Mechanistic insights into the selective electroreduction of carbon dioxide to ethylene on Cu_2O-derived copper catalysts. *J. Phys. Chem. C* **120**, 20058-20067 (2016).

18. Ren, D. et al. Mechanistic insights into the enhanced activity and stability of agglomerated Cu nanocrystals for the electrochemical reduction of carbon dioxide to n-propanol. *J. Phys. Chem. Lett.* **7**, 20-24 (2016).

19. Kim, D., Kley, C. S., Li, Y. & Yang, P. Copper nanoparticle ensembles for selective electroreduction of CO_2 to C_2–C_3 products. *Proc. Natl. Acad. Sci. U. S. A.* **114**, 10560-10565 (2017).

20. Rahaman, M., Dutta, A., Zanetti, A. & Broekmann, P. Electrochemical reduction of CO_2 into multicarbon alcohols on activated Cu mesh catalysts: an identical location (IL) study. *ACS Catal.* **7**, 7946-7956 (2017).

21. Jiang, K. et al. Metal ion cycling of Cu foil for selective C–C coupling in electrochemical CO_2 reduction. *Nat. Catal.* **1**, 111-119 (2018).

22. Zhuang, T.-T. et al. Steering post-C–C coupling selectivity enables high efficiency electroreduction of carbon dioxide to multi-carbon alcohols. *Nat. Catal.* **1**, 421-428 (2018).

23. Verdaguer-Casadevall, A. et al. Probing the active surface sites for CO reduction on oxide-derived copper electrocatalysts. *J. Am. Chem. Soc.* **137***,* 9808-9811 (2015).

24. Feng, X., Jiang, K., Fan, S. & Kanan, M. W. A direct grain-boundary-activity correlation for CO electroreduction on Cu nanoparticles. *ACS Cent. Sci.* **2**, 169-174 (2016).

25. Raciti, D. et al. Low-overpotential electroreduction of carbon monoxide using copper nanowires. *ACS Catal.* **7**, 4467-4472 (2017).

26. Jouny, M., Luc, W. & Jiao, F. High-rate electroreduction of carbon monoxide to multi-carbon products. *Nat. Catal.* **1**, 748-755 (2018).

27. Li, J. et al. Copper adparticle enabled selective electrosynthesis of n-propanol. *Nat. Commun.* **9**, 4614 (2018).

28. Zhuang, T.-T. et al. Copper nanocavities confine intermediates for efficient electrosynthesis of C3 alcohol fuels from carbon monoxide. *Nat. Catal.* **1**, 946-951 (2018).

29. Xiao, H., Cheng, T. & Goddard, W. A. Atomistic mechanisms underlying selectivities in C_1 and C_2 products from electrochemical reduction of CO on Cu (111). *J. Am. Chem. Soc.* **139**, 130-136 (2016).

30. Kuhl, K. P., Cave, E. R., Abram, D. N. & Jaramillo, T. F. New insights into the electrochemical reduction of carbon dioxide on metallic copper surfaces. *Energy Environ. Sci.* **5**, 7050-7059 (2012).

31. Wellendorff, J. et al. Density functionals for surface science: Exchange-correlation model

development with Bayesian error estimation. *Phys. Rev. B* **85**, 235149 (2012).

32. Perdew, J. P. et al. Restoring the density-gradient expansion for exchange in solids and surfaces. *Phys. Rev. Lett.* **100**, 136406 (2008).

33. Perdew, J. P. & Wang, Y. Accurate and simple analytic representation of the electron-gas correlation energy. *Phys. Rev. B* **45**, 13244 (1992)

34. Kozuch, S. & Shaik, S. How to conceptualize catalytic cycles? The energetic span model. *Acc. Chem. Res.* **44**, 101-110 (2011).

35. Rumble, J. R. *CRC Handbook of Chemistry and Physics* 99th edn (CRC Press, Cleveland, 2018).

36. Dinh, C.-T. et al. CO_2 electroreduction to ethylene via hydroxide-mediated copper catalysis at an abrupt interface. *Science* **360**, 783-787 (2018).
